# Dairy product consumption and incident prediabetes in Dutch middle-aged adults: the Hoorn Studies prospective cohort

**DOI:** 10.1007/s00394-021-02626-9

**Published:** 2021-07-10

**Authors:** Isabel A. L. Slurink, Nicolette R. den Braver, Femke Rutters, Nina Kupper, Tom Smeets, Petra J. M. Elders, Joline W. J. Beulens, Sabita S. Soedamah-Muthu

**Affiliations:** 1 Center of Research on Psychological and Somatic Disorders (CORPS), Department of Medical and Clinical Psychology, Tilburg University, PO Box 90153, 5000 LE Tilburg, The Netherlands; 2 Department of Epidemiology and Biostatistics, Amsterdam University Medical Centers, Vrije Universiteit Amsterdam, Amsterdam Public Health Research Institute, Amsterdam, The Netherlands; 3 Julius Center for Health Sciences and Primary Care, University Medical Center Utrecht, Utrecht, The Netherlands; 4 Institute for Food, Nutrition and Health, University of Reading, Reading, RG6 6AR UK

**Keywords:** Dairy, Impaired glucose metabolism, Prediabetes, Prospective cohort

## Abstract

**Purpose:**

Our aim was to investigate prospective associations of consumption of total dairy and dairy types with incident prediabetes in a Dutch population-based study.

**Methods:**

Two enrolment waves of the Hoorn Studies were harmonized, resulting in an analytic sample of 2262 participants without (pre-) diabetes at enrolment (mean age 56 ± 7.3 years; 50% male). Baseline dietary intake was assessed by validated food frequency questionnaires. Relative risks (RRs) were calculated between dairy, fermented dairy, milk, yogurt (all total/high/low fat), cream and ice cream and prediabetes. Additionally, substituting one serving/day of dairy types associated with prediabetes with alternative dairy types was analysed.

**Results:**

During a mean 6.4 ± 0.7 years of follow-up, 810 participants (35.9%) developed prediabetes. High fat fermented dairy, cheese and high fat cheese were associated with a 17% (RR 0.83, 95% CI 0.69–0.99, *p*_trend_ = 0.04), 14% (RR 0.86, 95% CI 0.73–1.02, *p*_trend_ = 0.04) and 21% (RR 0.79, 95% CI 0.66–0.94*, p*_trend_ = 0.01) lower risk of incident prediabetes, respectively, in top compared to bottom quartiles, after adjustment for confounders. High fat cheese consumption was continuously associated with lower prediabetes risk (RR_servings/day_ 0.94, 95% CI 0.88–1.00, *p* = 0.04). Total dairy and other dairy types were not associated with prediabetes risk in adjusted models, irrespective of fat content (RR ~ 1). Replacing high fat cheese with alternative dairy types was not associated with prediabetes risk.

**Conclusion:**

The highest intake of high fat fermented dairy, cheese and high fat cheese were associated with a lower risk of prediabetes, whereas other dairy types were not associated. Cheese seems to be inversely associated with type 2 diabetes risk, despite high levels of saturated fatty acids and sodium.

**Supplementary Information:**

The online version contains supplementary material available at 10.1007/s00394-021-02626-9.

## Introduction

Prediabetes is a condition characterized by blood glucose levels that are above the normal range, but still fall below the diagnostic threshold for type 2 diabetes [1].The prevalence of prediabetes is rapidly rising worldwide from 374 million in 2019 to an expected 548 million in 2045 [2]. People with prediabetes are at increased risk of developing type 2 diabetes (T2D) and cardiovascular diseases (CVD) [3, 4], but may reverse to normoglycaemia with lifestyle adaptation [5]. This emphasizes the need to identify modifiable risk factors that could prevent or reverse this condition. Suboptimal diet is causally linked to incidence of prediabetes and T2D, and majority of cases can be prevented by dietary modification [6, 7].

Dairy products are widely consumed and may provide considerable quantities of beneficial nutrients for metabolic health, including protein, minerals (calcium, magnesium, potassium) and vitamins (A, D, B_2_, B_12_), but also contain saturated fatty acids (SFAs) and sodium. A recent summary of meta-analyses reported dose–response relations of low fat dairy (RRs ranging from 0.88–0.98), yogurt (0.78–0.94) and cheese (0.80–1.00) with T2D, inconsistent results for milk (RRs 0.83–1.27), with considerable heterogeneity present between studies [8]. One possible explanation for heterogeneity, proposed by Hruby et al., could be differences in participant’s baseline glycaemic status [9], and the precise moment along the physiological progress of T2D at which specific dairy products modify risk is largely unknown. Therefore, studies aiming to elucidate associations between dairy and early-risk stages are warranted.

Only one prospective cohort study investigated the associations of dairy products with incident prediabetes, based on fasting plasma glucose (FPG) [9]. In the US FHS Offspring Cohort (*n* = 1867, 12-year follow-up), highest consumption of total, low fat and high fat dairy was associated with 39%, 32% and 25% lower prediabetes incidence, respectively, compared to lowest consumption, with nonlinear protective associations for milk and yogurt. In the French DESIR study (9-year follow-up), prediabetes and T2D were combined as one outcome, inhibiting interpretation of associations with prediabetes alone. The DESIR study observed an association between higher total dairy (except cheese) intake with lower hyperglycaemia incidence [10]. Furthermore, two studies investigating continuous outcomes of glucose metabolism showed no associations of any dairy types [11, 12], except for an association of higher fermented dairy with lower FPG and HbA1c in the Danish Inter99 study [11]. Evidence from cross-sectional studies indicated that mainly higher fermented and skimmed dairy intake were associated with lower prediabetes risk [13–15], with one study also reporting associations for higher non-fermented and high fat dairy intake and higher prediabetes risk [14]. Thus, although there are some indications for beneficial associations of dairy consumption on prediabetes risk, associations are highly heterogeneous, partly underlined by different definitions of prediabetes outcomes and large variations in dairy consumption habits, advocating the need for country and region-specific prospective data.

Therefore, this study aimed to investigate prospective associations between consumption of total dairy and dairy types with incident prediabetes.

## Methods

### Study population

This study used data of the Hoorn Studies, a prospective population-based cohort study with the first enrolment wave in 1989–1992 (Hoorn Study 1, HS1) and a second wave in 2006–2007 (HS2). The aim was to study prevalence and risk factors for disturbances in glucose metabolism and T2D. Both enrolment waves were similar in design, population characteristics and questionnaires [16], and could, therefore, be harmonized to increase sample size and study power. Furthermore, this harmonization resulted in increased variation in dairy product intake and inclusion of more up-to-date information. People from the general population were recruited, aged 50–75 years in the HS1 and 40–65 years in the HS2 at time of inclusion. Follow-up measurements were performed between the years 1996–1998 in the HS1 and 2013–2015 in the HS2. Visits took place at the Diabetes Care Center in the city of Hoorn, the Netherlands. Written informed consent was obtained from all participants. The study was approved by the Ethics Committee of the Amsterdam University Medical Centers, location VUMC.

From participants with follow-up data available (*n* = 3245), we excluded participants with prediabetes (*n* = 557) or T2D at baseline (*n* = 229) or follow-up (*n* = 528) (Supplemental Fig. 1). Exclusion of prediabetes was based on FPG between 6.1–7.0 mmol/L, 2 h plasma glucose (2hPG) between 7.8 and 11.0 mmol/L and/or HbA1c levels between 6.0 and 6.5% [1, 17]. Exclusion of T2D was based on diagnosis by a general practitioner, diabetes medication user and/or an FPG ≥ 7.0 mmol/L, 2hPG ≥ 11.1 mmol/L, or HbA1c ≥ 6.5%. Other exclusion criteria were self-reported history of T2D prior to baseline (*n* = 59), extreme energy intake (top and bottom 0.5%) (*n* = 34) or missing information on dairy intake (*n* = 42), and/or missing data on prediabetes at follow-up (*n* = 9). After exclusion, the analytic sample consisted of 2262 participants.

### Assessment of dairy consumption

Baseline dietary intake was assessed with a 92-item food frequency questionnaire (FFQ) in the HS1 and a 104-item FFQ in the HS2. The HS1 FFQ was validated against a dietary history in 74 males and females, and was valid for ranking individuals according to energy intake (*r* = 0.72), and main nutrients in dairy products; animal protein (*r* = 0.82), SFAs (*r* = 0.73) and calcium (*r* = 0.75) [18]. The HS2 FFQ was validated against actual energy intake in controlled feeding trials for energy intake (*r* = 0.82) [19] and validated against three 24-h recalls for animal protein (*r* = 0.49), SFA (*r* = 0.44) and calcium (*r* = 0.56) [20].

Participants were asked to report their usual frequency of consumption, serving size and preparation in the past year. Seasonal variations in milk consumption were assessed with separate questions for winter and summer intakes. Participants completed the questionnaire at home, and checked for completeness by a trained dietician. Intake (gram/day) per FFQ item was calculated using the Dutch food composition table (NEVO) 1989/1990 for HS1 and the NEVO 2006 for HS2. FFQ items were combined and categorized as total dairy, fermented dairy, and by subtypes milk (all types and regular milk), yogurt, cheese, cream and ice cream (Table 1). Each dairy category was further divided into low fat (liquid products, ≤ 2%; cheese ≤ 20%) and high fat (liquid products, > 2%; cheese > 20%). Intakes were converted to servings/day according to Dutch standard serving sizes: milk, 200 mL; yogurt, 150 mL; cheese, 20 g; cream, 3 g; ice cream, 100 g (https://portie-online.rivm.nl/). In the total dairy category, a serving of liquid dairy products was defined as 200 mL and a serving of cheese as 20 g. Because two different FFQs were used, dietary intakes of food groups and dairy types stratified by enrolment wave are reported in Supplementary Table 1.

**Table 1:** Individual dairy products included and grouping for the present analysis of the Hoorn Studies

Dairy product	Included dairy types
Total dairy	All dairy products
High fat dairy	All high fat dairy products
Low fat dairy	All low fat dairy products
Fermented dairy	
High fat^1^	Full fat yogurt, full fat fruit yogurt, full fat curd, high fat cheese, full fat luxury cheese
Low fat^2^	Semi-skimmed yogurt, skimmed yogurt, skimmed fruit yogurt, semi-skimmed curd, skimmed curd, semi-skimmed fruit curd, skimmed fruit curd, low fat cheese, low fat luxury cheese
Milk, all types	
High fat	Full fat milk, full fat chocolate milk, milk powder, full fat milk added to the coffee, drinking yogurt^3^, fruit flavoured milk^3^
Low fat	Semi-skimmed milk, skimmed milk, buttermilk, semi-skimmed chocolate milk, skimmed chocolate milk, semi-skimmed milk added to the coffee, skimmed milk added to the coffee, semi-skimmed fruit milk
Regular milk	
High fat	Full fat milk
Low fat	Semi-skimmed milk, skimmed milk, buttermilk
Yogurt	
High fat^1^	Full fat yogurt, full fat fruit yogurt
Low fat^4^	Semi-skimmed yogurt, skimmed yogurt, skimmed fruit yogurt
Cheese	
High fat	HS1: regular cheese, cheese cubes; HS2: 40 + cheese (e.g. Edam), 48 + cheese (e.g. Gouda, cheddar, cheese spread, goat cheese), full fat luxury cheese (e.g. cream brie, cream cheese, mon chou), cheese cubes, grated cheese, feta, cheese fondue
Low fat	HS1: skimmed cheese; HS2: 20 + and 30 + cheese (e.g. cheese spread, cottage cheese), low fat luxury cheese (e.g. brie, goat cheese)
Cream (high fat)	Whipped cream, coffee cream, semi-skimmed coffee cream, sour cream, crème fraiche, cooking cream
Ice cream (high fat)	Ice cream

*HS1* Hoorn Studies 1 (first enrolment wave), *HS2* Hoorn Studies 2 (second enrolment wave)

^1^Includes oatmeal porridge, rice porridge and full fat custard in HS2

^2^Includes buttermilk in HS1, buttermilk porridge and skimmed custard in HS2

^3^Only assessed in HS2

^4^Includes semi-skimmed curd, skimmed curd, semi-skimmed fruit curd, skimmed fruit curd, buttermilk porridge and skimmed custard in HS2

### Outcome assessment

At all study visits, bloods samples were drawn to determine FPG, 2hPG levels after a 75-g oral glucose tolerance test (OGTT) and HbA_1c_ levels, except at the HS2 follow-up visit, where no OGTT was conducted and HbA_1c_ was measured in fasting capillary blood samples obtained using a blood spot card. FPG and 2hPG levels were determined using the glucose dehydrogenase method (Merck, Darmstadt, Germany). In the HS1, HbA_1c_ was determined by ion-exchange high-performance liquid chromatography with a Modular Diabetes Monitoring System (Bio-Rad, Veenendaal, The Netherlands). In the HS2 at baseline, HbA_1c_ levels were assessed using standardized reverse-phase cation-exchange chromatography (HA 8160 analyzer; Menarini, Florence, Italy). In the HS2 at follow-up, HbA_1c_ levels were derived from blood spot cards, using thermo immunoturbidimetry according to a validated protocol [21]. Prediabetes at follow-up was defined according to the diagnostic criteria of the World Health Organization of 2006 [1], complemented with the HbA_1c_ cut-offs values proposed by the 2009 international expert committee for prediabetes [17], with FPG between 6.1–7.0 mmol/L, 2hPG between 7.8–11.0 mmol/L and/or HbA_1c_ levels between 6.0–6.5%.

### Other variables

The self-administered baseline questionnaire for both enrolment waves included questions on socio-demographic, lifestyle and clinical factors. Responses were verified in a personal interview. Smoking status was categorized as current, former, or never. Highest educational level was obtained in eight levels, which were subsequently categorized to low (no education or primary school), middle (secondary education) and high (tertiary education). Moderate physical activity in hours/week was assessed using the SQUASH questionnaire, for which the Spearman correlation for overall reproducibility was 0.58 in 50 participants compared to an activity monitor [22]. The activities included sports, bicycling, gardening, walking, doing chores and housekeeping. Alcohol intake was categorized as non-drinker, ≤ 10, 10–30 and ≥ 30 g/day. Family history of diabetes was defined as having at least a grandparent, parent, sibling or child with diabetes.

Physical measurements were performed at baseline. BMI was calculated as weight divided by height squared (kg/m^2^), and categorized as < 25 kg/m^2^, 25–30 kg/m^2^ and ≥ 30 kg/m^2^. Blood pressure was measured on the right arm with a random-zero sphygmomanometer (Hawksley–Gelman Ltd, Lancing, United Kingdom) while participants were sitting. Plasma levels of total cholesterol, triglycerides, high-density lipoprotein (HDL) were measured in fasting blood samples by enzymatic techniques (Boehringer-Mannheim, Mannheim, Germany) and low-density lipoprotein (LDL) was calculated using the Friedewald formula (except for participants with triglycerides > 4.55 mmol/L) [23].

### Statistical analysis

Statistical analyses were performed using R version 3.6.2 (R Foundation for Statistical Computing, Vienna, Austria). Baseline characteristics are displayed as means ± SD, medians (IQR) or percentages for the total study population and in quartiles of total dairy intake. Missing values in confounding variables (for 6% of participants, highest 2% for physical activity) were imputed using multiple imputation (*n* = 10).

Poisson regression with robust variance was used to examine associations between dairy product intakes and prediabetes, because of the high incidence of prediabetes (35.9%), in which case the odds ratio overestimates the strength of the association [24]. RRs with 95% CIs were calculated for quartiles of dairy intake (reference lowest) and on a continuous scale (servings/day). Dairy products for which many participants reported no intake were divided in a non-consumer category (reference) and consumers in tertiles. Linear trend across intake range categories were assessed by including median values of each category as a continuous variable in the model. Linearity was assumed in all models, as no indications for non-linearity presented assessed by adding a quadratic term to model 3. Regression coefficients for each of the imputed datasets were pooled.

Confounder models were constructed based on literature [25] and on distributions of baseline characteristics across quartiles. Model 1 included age, sex, follow-up duration and enrolment wave. Model 2 additionally adjusted for energy intake, education, smoking, physical activity, alcohol consumption and family history of diabetes. Model 3 additionally adjusted for food groups associated with T2D including intakes of fruit, vegetables, tea, coffee, grains (whole and refined), meat (processed and red) and sugar-sweetened beverages [26, 27]. BMI, blood pressure (systolic and diastolic) and LDL cholesterol were added separately in model 4 because of their potential mediating or otherwise confounding effect. We checked for effect modification by enrolment wave by including an interaction term in model 3 and stratified associations by enrolment wave to assess if associations differed for each wave of the Hoorn Studies. Furthermore, effect modification by age, sex and BMI was examined, and associations were stratified in case of significance.

We provided a supplementary baseline table stratified by the dairy types that were significantly associated with prediabetes in the main analyses, to examine confounding of associations by healthy lifestyle. Furthermore, we examined substituting one serving/day of significant dairy types with alternative dairy types in model 3. Models included a total dairy intake variable (servings/day), all individual dairy types (servings/day) except for the dairy type to be substituted, and energy intake. The estimated RR for each alternative dairy type can be interpreted as the RR for substitution of a daily serving of the alternative dairy type for a daily serving of the excluded dairy type [28].

A series of sensitivity analyses were conducted using model 3. First, the independence of the associations of specific dairy types was tested by mutually adjusting for intake of other dairy types. Second, we repeated the analysis excluding participants with self-reported CVD (*n* = 261). Third, we repeated the analysis in ‘normal energy reporters’ only, identified using the Goldberg method [29]. For this method, the basal metabolic rate (BMR) was calculated for each participant using Schofield equations specifically for age and sex categories based on weight [30]. Following, the ratio of energy intake (EI) and BMR was calculated. Using the Goldberg cut-offs described by Black et al., participants with EI:BMR < 1.08 were classified as under-reporters, participants with 1.08 ≤ EI:BMR ≤ 2.22 were classified as ‘normal reporters’ and those with EI:BMR > 2.22 were defined as ‘over-reporters’ [29]. Lastly, to address possible misclassification in prediabetes defined at baseline, we repeated analyses including participants with prediabetes at baseline (*n* = 557, final sample *n* = 2661).

## Results

### Participant characteristics

**Fig. 1:**
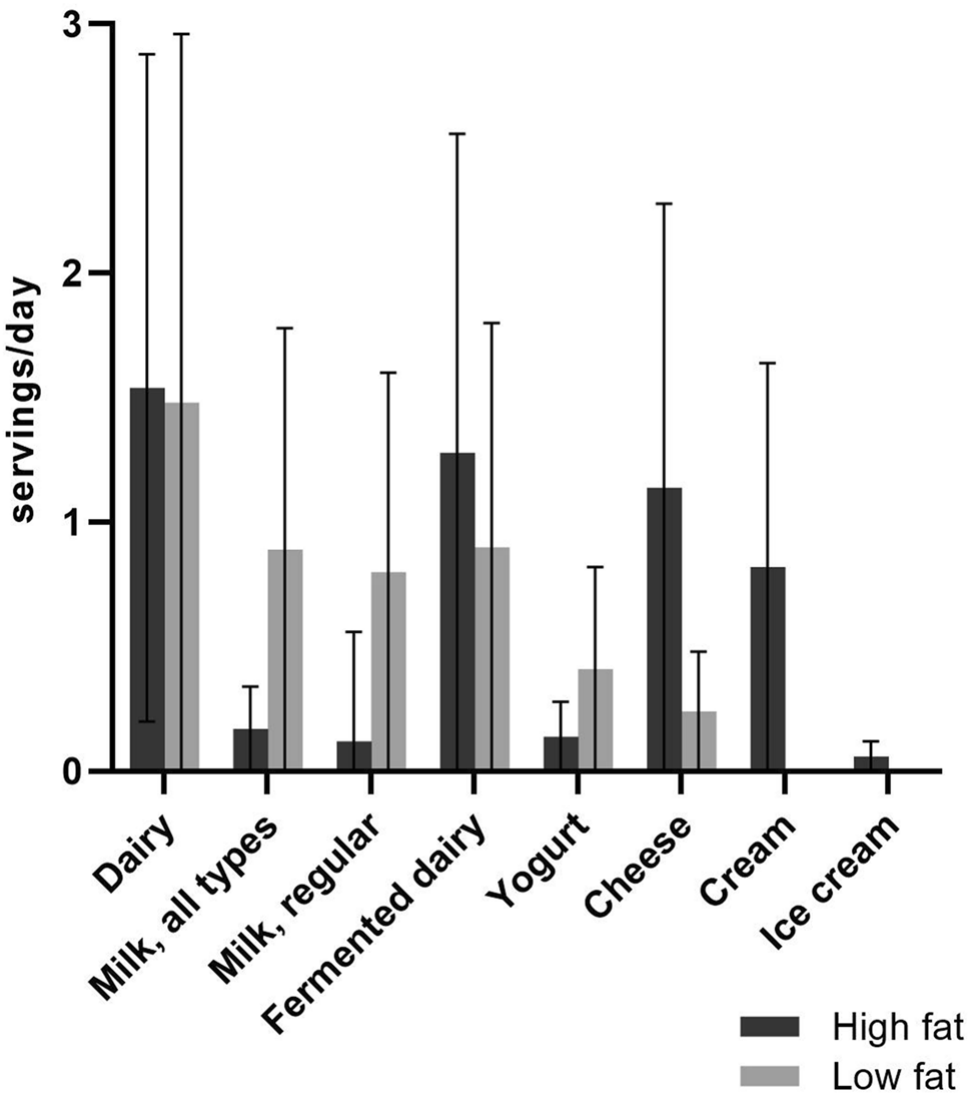
Mean dairy consumption in the Hoorn Studies (*n* = 2262)

The mean age of the study population was 55.9 ± 7.3 years, 50% were male and 22% were current smokers (Table 2, by enrolment wave Supplemental Table 1). The mean BMI was 25.7 ± 3.4 kg/m^2^ and 10% of participants were obese (BMI ≥ 30 kg/m^2^). The mean dairy intake was 3.0 ± 1.7 servings/day (357 ± 237 g/day) (Fig. 1). Participants in the top (3.9–15.4 servings/day) compared to the bottom quartile (0–1.8 servings/day) of dairy intake were more often male (54% vs 43%), with low education (16% vs 8%), more physically active (median (IQR): 9.0 (5.1–13.7) vs 6.5 (3.5–10.5) hours/week) and had higher LDL cholesterol levels (4.2 ± 1.1 vs 3.5 ± 1.0 mmol/L). With increasing dairy intake over the quartiles, energy, calcium, fruit and processed meat intakes were higher and vegetable and alcohol intakes were lower. Participants with complete follow-up data (*n* = 3245*)* were similar to participants lost-to-follow-up (*n* = 2046) with regard to age (56.9 ± 7.6 vs 58.0 ± 9.0 years), sex (51% vs 49% male), physical activity (7.5 IQR 4.0–12.3 vs 7.0 IQR 3.5–12.7 h/week), BMI (26.3 ± 3.6 vs 26.6 ± 4.1 kg/m^2^) and fasting glucose (5.6 ± 1.0 vs 5.8 ± 1.6 mmol/L) (Supplemental Table 2). Participants lost-to-follow-up were slightly more often lower educated (22% vs 15%), current smoker (31% vs 23%) and had a lower alcohol intake (median 5.0 IQR 0.0–15.3 vs 7.2 IQR 2.0–17.2).

**Table 2:** Baseline characteristics and dietary intakes of participants of the Hoorn Studies according to quartiles of total dairy intake (*n* = 2262)

	*N* missing	Total population	Q1	Q2	Q3	Q4
		(*n* = 2262)	(*n* = 562)	(*n* = 573)	(*n* = 561)	(*n* = 566)
Dairy intake (servings/day)						
Range			0–1.8	1.8–2.7	2.7–3.9	3.9–15.4
Median		2.7	1.3	2.3	3.3	5.0
Follow-up time (year)		6.4 ± 0.7	6.6 ± 0.7	6.4 ± 0.7	6.4 ± 0.7	6.2 ± 0.6
Sex (men)		50% (1132)	43% (243)	52% (296)	52% (289)	54% (304)
Age (year)		55.9 ± 7.3	54.1 ± 7.1	55.6 ± 7.3	56.6 ± 7.5	57.3 ± 7.1
Education level	22					
Low		13% (299)	8% (46)	15% (84)	14% (79)	16% (90)
Middle		58% (1310)	60% (336)	54% (309)	57% (317)	61% (348)
High		28% (631)	31% (172)	30% (174)	28% (159)	22% (126)
Smoking	10					
Current		22% (493)	20% (115)	23% (131)	22% (124)	22% (123)
Previous (> 2 months ago)		38% (851)	40% (226)	37% (211)	37% (208)	36% (206)
Never		40% (908)	38% (216)	40% (228)	41% (228)	42% (236)
Cigarette years	663	210 (9–480)	290 (100–560)	220 (30–470)	180 (0–450)	160 (0–460)
Alcohol intake	1					
0 g/day		18% (401)	15% (87)	18% (103)	17% (97)	20% (114)
≤ 10 g/day		42% (959)	42% (237)	43% (246)	40% (226)	44% (250)
10–30 g/day		30% (678)	30% (170)	31% (178)	32% (179)	27% (151)
≥ 30 g/day		10% (223)	12% (68)	8% (45)	11% (59)	9% (51)
Family history diabetes mellitus	11	24% (553)	25% (141)	27% (152)	21% (119)	25% (141)
Physical activity, moderate intensity, hours/week	47	7.5 (4.2–12.0)	6.5 (3.5–10.5)	7.0 (3.8–12.0)	7.8 (4.5–12.6)	9.0 (5.1–13.7)
BMI (kg/m^2^)	4	25.7 ± 3.4	25.9 ± 3.5	25.5 ± 3.2	25.5 ± 3.3	26.1 ± 3.4
Fasting glucose (mmol/L)	7	5.3 ± 0.4	5.3 ± 0.4	5.3 ± 0.4	5.2 ± 0.4	5.2 ± 0.4
Systolic blood pressure (mmHg)	2	130 ± 17	130 ± 16	130 ± 18	130 ± 17	130 ± 18
Diastolic blood pressure (mmHg)	5	78 ± 11	78 ± 11	78 ± 11	79 ± 10	80 ± 10
Antihypertensive medication use		13% (304)	16% (92)	12% (70)	13% (73)	12% (69)
LDL cholesterol (mmol/L)	6	3.8 ± 1.1	3.5 ± 1.0	3.8 ± 1.1	3.9 ± 1.1	4.2 ± 1.1
Lipid lowering medication		5% (108)	9% (50)	4% (22)	3% (18)	3% (18)
Dietary intake						
Energy intake (kcal/day)		2100 ± 600	1900 ± 540	2100 ± 530	2200 ± 570	2400 ± 620
DHD15-index score		70 ± 14	66 ± 14	71 ± 14	72 ± 14	70 ± 13
Fruit (g/day)		200 ± 140	160 ± 130	190 ± 130	200 ± 130	240 ± 150
Vegetables (g/day)		150 ± 85	170 ± 91	150 ± 83	150 ± 86	140 ± 75
Grain (g/day)	5	200 ± 95	200 ± 95	200 ± 92	200 ± 96	200 ± 96
Red meat (g/day)	1	34 ± 23	37 ± 23	35 ± 22	33 ± 23	31 ± 23
Processed meat (g/day)		46 ± 33	37 ± 30	44 ± 32	47 ± 33	54 ± 35
Lean fish (g/day)		11 ± 13	11 ± 12	11 ± 13	11 ± 12	12 ± 14
Fatty fish (g/day)		5.0 ± 8.4	4.9 ± 7.2	5.4 ± 10.6	4.7 ± 7.4	4.9 ± 8.1
Coffee (g/day)		500 ± 270	450 ± 290	490 ± 270	520 ± 250	540 ± 270
Tea (g/day)	3	280 ± 260	270 ± 290	280 ± 250	290 ± 250	300 ± 260
Fruit juice (g/day)	3	60 ± 95	69 ± 110	60 ± 89	54 ± 83	55 ± 98
Sugar-sweetened beverages (g/day)		110 ± 140	130 ± 160	110 ± 140	100 ± 130	110 ± 150
Saturated fat (en%)		14.6 ± 3.7	12.6 ± 3.3	14.2 ± 3.3	15.2 ± 3.4	16.6 ± 3.5
Protein (en%)		14.5 ± 2.4	13.8 ± 2.3	14.3 ± 2.3	14.6 ± 2.2	15.4 ± 2.5
Calcium (g/day)		1000 ± 370	650 ± 180	880 ± 160	1100 ± 160	1500 ± 300

Variables are displayed as means ± SD for normally distributed continuous variables, medians (IQR) for non-normally distributed continuous variables or % (*n*) for categorical variables

*BMI* body mass index, *DHD15-index* Dutch Healthy Diet 2015 index score [56], *HS1* Hoorn Studies 1 (first enrolment wave), *LDL* low-density lipoprotein

### Dairy intake and incident prediabetes

During a mean follow-up duration of 6.4 ± 0.7 years, 811 out of 2262 participants developed prediabetes (35.9%). High fat fermented dairy intake was significantly associated with lower prediabetes risk in model 3 (RR_Q4vsQ1_: 0.83, 95% CI 0.69–0.99, *p*_trend_ = 0.04) (Table 3). High fat fermented dairy intake mainly consisted of cheese intake (63%), which was marginally significantly associated with lower risk of prediabetes (RR top vs bottom quartile: 0.86, 95% CI 0.73–1.02, *p*_trend_ = 0.04). Specifically, higher intake of high fat cheese (52% of high fat fermented dairy, 83% of total cheese intake) was significantly associated with lower prediabetes risk (RR_Q4vsQ1_: 0.79, 95% CI 0.66–0.94, *p*_trend_ = 0.006). High fat cheese was the only dairy type continuously associated with prediabetes (RR_servings/day_: 0.94, 95% CI 0.88–1.00, *p* = 0.04). Total dairy, fermented dairy, milk, regular milk, yogurt, cream and ice cream intake both in quartiles and continuously were not associated with risk of prediabetes in multivariate adjusted models (RR ~ 1). Further adjustment for BMI, LDL cholesterol and blood pressure in model 4 did not change the associations.

**Table 3:** Risk ratios (95% confidence interval) for the association between dairy intake and incidence of prediabetes, the Hoorn Studies (*n* = 2262)

	Continuous^1^	Intake range categories				*p*_trend_
	RR (95% CI)		RR (95% CI)	RR (95% CI)	RR (95% CI)	
Total dairy		Q1	Q2	Q3	Q4	
n/N		216/562	216/573	183/561	196/566	
Median		1.3	2.3	3.3	5.0	
Model 1	0.98 (0.95–1.02)	1	1.00 (0.86–1.16)	0.88 (0.75–1.03)	0.96 (0.82–1.14)	0.49
Model 2	0.98 (0.94–1.02)	1	1.00 (0.86–1.17)	0.87 (0.73–1.02)	0.95 (0.80–1.14)	0.41
Model 3	0.98 (0.94–1.02)	1	1.00 (0.86–1.16)	0.87 (0.74–1.03)	0.95 (0.79–1.13)	0.38
Model 4	0.97 (0.93–1.01)	1	1.01 (0.87–1.18)	0.88 (0.74–1.04)	0.93 (0.78–1.12)	0.30
High fat dairy		Q1	Q2	Q3	Q4	
n/N		217/565	216/567	198/563	180/567	
Median		0.1	0.9	1.7	3.1	
Model 1	0.97 (0.92–1.01)	1	1.01 (0.87–1.18)	0.95 (0.81–1.12)	0.88 (0.74–1.05)	0.11
Model 2	0.95 (0.91–1.00)	1	**1.00 (0.86–1.16)**	**0.92 (0.78–1.08)**	**0.84 (0.70–1.01)**	**0.04**
Model 3	0.96 (0.91–1.01)	1	1.01 (0.87–1.17)	0.94 (0.79–1.11)	0.85 (0.71–1.03)	0.06
Model 4	0.95 (0.91–1.00)	1	1.00 (0.86–1.16)	0.94 (0.80–1.11)	0.85 (0.71–1.03)	0.07
Low fat dairy		Q1	Q2	Q3	Q4	
n/N		203/566	210/561	208/576	190/559	
Median		0.2	0.9	1.6	2.8	
Model 1	1.01 (0.96–1.06)	1	1.03 (0.89–1.21)	1.00 (0.86–1.16)	0.95 (0.81–1.12)	0.46
Model 2	1.01 (0.97–1.06)	1	1.06 (0.91–1.24)	1.02 (0.87–1.19)	0.98 (0.83–1.15)	0.67
Model 3	1.01 (0.96–1.06)	1	1.06 (0.91–1.23)	1.01 (0.87–1.18)	0.96 (0.82–1.13)	0.48
Model 4	1.00 (0.96–1.05)	1	1.06 (0.91–1.23)	1.00 (0.86–1.17)	0.94 (0.80–1.11)	0.35
Total fermented dairy		Q1	Q2	Q3	Q4	
n/N		224/570	188/562	205/566	194/564	
Median		0.7	1.5	2.4	3.7	
Model 1	0.98 (0.94–1.02)	1	0.86 (0.73–1.00)	0.94 (0.81–1.10)	0.94 (0.80–1.10)	0.73
Model 2	0.98 (0.94–1.02)	1	0.87 (0.75–1.02)	0.95 (0.81–1.10)	0.94 (0.79–1.11)	0.68
Model 3	0.98 (0.94–1.03)	1	0.88 (0.75–1.03)	0.95 (0.81–1.11)	0.95 (0.80–1.13)	0.81
Model 4	0.98 (0.93–1.02)	1	0.89 (0.76–1.04)	0.95 (0.81–1.11)	0.94 (0.79–1.11)	0.65
High fat fermented dairy		Q1	Q2	Q3	Q4	
n/N		226/571	188/513	219/613	178/565	
Median		0.0	0.7	1.4	2.6	
Model 1	0.96 (0.91–1.01)	1	0.94 (0.81–1.09)	0.93 (0.80–1.09)	0.85 (0.72–1.00)	0.05
Model 2	0.94 (0.89–1.00)	1	**0.93 (0.79–1.08)**	**0.91 (0.78–1.07)**	**0.81 (0.68–0.97)**	**0.02**
Model 3	0.95 (0.90–1.01)	1	**0.94 (0.80–1.09)**	**0.93 (0.79–1.08)**	**0.83 (0.69–0.99)**	**0.04**
Model 4	0.95 (0.89–1.00)	1	0.94 (0.80–1.10)	0.93 (0.80–1.09)	0.82 (0.68–0.98)	0.03
Low fat fermented dairy		Q1	Q2	Q3	Q4	
n/N		207/565	204/566	203/557	197/574	
Median		0.0	0.4	0.9	1.9	
Model 1	1.02 (0.97–1.09)	1	0.97 (0.83–1.13)	0.97 (0.83–1.13)	0.95 (0.81–1.11)	0.55
Model 2	1.03 (0.97–1.10)	1	0.99 (0.84–1.15)	0.99 (0.84–1.15)	0.97 (0.83–1.14)	0.73
Model 3	1.03 (0.97–1.10)	1	0.99 (0.85–1.16)	0.98 (0.84–1.15)	0.96 (0.82–1.13)	0.64
Model 4	1.03 (0.97–1.09)	1	0.99 (0.85–1.16)	0.99 (0.84–1.15)	0.96 (0.82–1.12)	0.57
Total milk, all types		Q1	Q2	Q3	Q4	
n/N		197/562	206/570	222/566	186/564	
Median		0.1	0.6	1.2	2.1	
Model 1	1.01 (0.95–1.07)	1	1.04 (0.89–1.21)	1.15 (0.99–1.34)	1.00 (0.85–1.19)	0.83
Model 2	1.01 (0.95–1.08)	1	1.05 (0.90–1.22)	1.16 (1.00–1.35)	1.01 (0.85–1.20)	0.78
Model 3	1.00 (0.94–1.06)	1	1.02 (0.87–1.19)	1.13 (0.97–1.31)	0.96 (0.81–1.15)	0.79
Model 4	0.99 (0.94–1.06)	1	1.02 (0.88–1.19)	1.14 (0.98–1.32)	0.96 (0.81–1.14)	0.81
High fat milk, all types		Zero	T1	T2	T3	
n/N		497/1,339	97/307	82/248	135/368	
Median		0.0	0.0	0.2	0.5	
Model 1	1.05 (0.93–1.18)	1	0.89 (0.74–1.06)	0.99 (0.80–1.22)	1.08 (0.91–1.28)	0.27
Model 2	1.03 (0.92–1.16)	1	0.90 (0.76–1.08)	1.00 (0.81–1.23)	1.06 (0.89–1.25)	0.43
Model 3	1.01 (0.89–1.14)	1	0.91 (0.76–1.09)	1.01 (0.82–1.24)	1.04 (0.87–1.24)	0.55
Model 4	1.02 (0.91–1.15)	1	0.91 (0.76–1.09)	1.01 (0.82–1.24)	1.06 (0.89–1.26)	0.42
Low fat milk, all types		Q1	Q2	Q3	Q4	
n/N		203/569	185/520	198/499	225/674	
Median		0.0	0.4	0.8	1.7	
Model 1	1.00 (0.94–1.07)	1	1.01 (0.86–1.18)	1.06 (0.91–1.22)	1.00 (0.84–1.18)	0.96
Model 2	1.01 (0.94–1.07)	1	1.03 (0.88–1.21)	1.07 (0.93–1.24)	1.02 (0.86–1.21)	0.79
Model 3	0.99 (0.93–1.06)	1	1.01 (0.86–1.19)	1.04 (0.90–1.21)	0.98 (0.83–1.17)	0.88
Model 4	0.99 (0.93–1.05)	1	1.01 (0.86–1.18)	1.03 (0.89–1.20)	0.98 (0.82–1.15)	0.80
Total regular milk		Q1	Q2	Q3	Q4	
n/N		183/534	187/521	290/755	151/452	
Median		0.0	0.3	1.0	2.1	
Model 1	1.02 (0.96–1.08)	1	1.08 (0.92–1.28)	1.14 (0.98–1.32)	1.07 (0.89–1.29)	0.41
Model 2	1.02 (0.96–1.09)	1	1.09 (0.93–1.29)	1.15 (0.99–1.33)	1.07 (0.89–1.30)	0.42
Model 3	1.00 (0.94–1.07)	1	1.07 (0.91–1.26)	1.11 (0.95–1.28)	1.02 (0.85–1.24)	0.79
Model 4	1.00 (0.94–1.07)	1	1.07 (0.91–1.25)	1.11 (0.96–1.29)	1.01 (0.84–1.23)	0.83
High fat regular milk		Zero	T1	T2	T3	
n/N		711/1,989	30/91	34/82	36/100	
Median		0.0	0.2	0.7	1.8	
Model 1	1.04 (0.92–1.17)	1	0.98 (0.72–1.32)	1.19 (0.91–1.55)	1.07 (0.82–1.41)	0.41
Model 2	1.02 (0.90–1.16)	1	0.95 (0.70–1.28)	1.17 (0.90–1.53)	1.04 (0.79–1.37)	0.56
Model 3	1.00 (0.88–1.13)	1	0.94 (0.69–1.27)	1.18 (0.90–1.54)	0.99 (0.75–1.31)	0.81
Model 4	1.01 (0.89–1.15)	1	0.94 (0.70–1.27)	1.19 (0.90–1.57)	1.03 (0.78–1.36)	0.61
Low fat regular milk		Q1	Q2	Q3	Q4	
n/N		212/586	170/512	236/598	193/566	
Median		0.0	0.3	0.7	1.9	
Model 1	1.01 (0.95–1.08)	1	0.94 (0.80–1.11)	1.12 (0.97–1.30)	0.99 (0.85–1.16)	0.87
Model 2	1.02 (0.96–1.09)	1	0.95 (0.80–1.12)	1.13 (0.98–1.31)	1.00 (0.85–1.17)	0.81
Model 3	1.01 (0.94–1.07)	1	0.93 (0.79–1.09)	1.10 (0.95–1.28)	0.97 (0.82–1.13)	0.87
Model 4	1.00 (0.94–1.07)	1	0.91 (0.78–1.07)	1.10 (0.95–1.27)	0.95 (0.81–1.12)	0.79
Total yogurt		Q1	Q2	Q3	Q4	
n/N		201/564	196/538	205/583	209/577	
Median		0.0	0.4	0.6	1.0	
Model 1	1.02 (0.91–1.14)	1	1.00 (0.86–1.18)	0.99 (0.85–1.15)	1.01 (0.86–1.18)	0.98
Model 2	1.05 (0.93–1.17)	1	1.03 (0.88–1.21)	1.01 (0.86–1.18)	1.04 (0.89–1.22)	0.69
Model 3	1.04 (0.93–1.17)	1	1.05 (0.89–1.23)	1.02 (0.87–1.20)	1.04 (0.88–1.21)	0.73
Model 4	1.06 (0.94–1.18)	1	1.04 (0.89–1.22)	1.03 (0.88–1.21)	1.05 (0.90–1.23)	0.56
High fat yogurt		Zero	T1	T2	T3	
n/N		550/1,575	86/229	88/219	87/239	
Median		0.0	0.1	0.3	0.8	
Model 1	1.07 (0.91–1.27)	1	1.08 (0.90–1.29)	1.14 (0.95–1.36)	1.08 (0.90–1.29)	0.25
Model 2	1.08 (0.91–1.29)	1	1.09 (0.91–1.31)	1.16 (0.97–1.39)	1.08 (0.90–1.30)	0.24
Model 3	1.09 (0.92–1.29)	1	1.10 (0.92–1.32)	1.19 (0.99–1.43)	1.09 (0.91–1.32)	0.19
Model 4	1.12 (0.94–1.34)	1	1.08 (0.90–1.29)	1.20 (1.01–1.44)	1.11 (0.92–1.34)	0.13
Low fat yogurt		Zero	T1	T2	T3	
n/N		256/724	223/604	152/416	180/518	
Median		0.0	0.2	0.6	0.9	
Model 1	0.99 (0.88–1.12)	1	1.01 (0.87–1.18)	1.00 (0.85–1.18)	0.97 (0.84–1.13)	0.70
Model 2	1.01 (0.90–1.15)	1	1.04 (0.89–1.21)	1.02 (0.86–1.20)	1.00 (0.86–1.17)	0.94
Model 3	1.01 (0.89–1.14)	1	1.04 (0.90–1.21)	1.03 (0.87–1.21)	0.99 (0.85–1.16)	0.82
Model 4	1.01 (0.89–1.14)	1	1.02 (0.88–1.19)	1.02 (0.87–1.20)	0.99 (0.85–1.15)	0.89
Total cheese		Q1	Q2	Q3	Q4	
n/N		215/563	216/561	202/581	178/557	
Median		0.3	0.9	1.5	2.6	
Model 1	0.96 (0.91–1.01)	1	**1.01 (0.87–1.17)**	**0.91 (0.78–1.07)**	**0.87 (0.74–1.02)**	**0.04**
Model 2	0.94 (0.89–1.00)	1	**1.01 (0.87–1.17)**	**0.90 (0.77–1.04)**	**0.84 (0.71–0.99)**	**0.02**
Model 3	0.95 (0.90–1.01)	1	**1.02 (0.88–1.19)**	**0.90 (0.77–1.05)**	**0.86 (0.73–1.02)**	**0.04**
Model 4	0.94 (0.89–1.00)	1	**1.02 (0.88–1.18)**	**0.89 (0.76–1.03)**	**0.84 (0.71–1.00)**	**0.02**
High fat cheese		Q1	Q2	Q3	Q4	
n/N		223/558	225/600	188/532	175/572	
Median		0.0	0.6	1.2	2.3	
Model 1	**0.94 (0.89–1.00)**	**1**	**0.96 (0.83–1.11)**	**0.92 (0.79–1.08)**	**0.81 (0.69–0.95)**	**0.009**
Model 2	**0.93 (0.88–0.99)**	**1**	**0.94 (0.82–1.09)**	**0.90 (0.77–1.06)**	**0.77 (0.65–0.92)**	**0.002**
Model 3	**0.94 (0.88–1.00)**	**1**	**0.95 (0.82–1.10)**	**0.91 (0.77–1.07)**	**0.79 (0.66–0.94)**	**0.006**
Model 4	**0.93 (0.88–0.99)**	**1**	**0.95 (0.82–1.11)**	**0.91 (0.77–1.07)**	**0.78 (0.65–0.93)**	**0.003**
Low fat cheese		Zero	T1	T2	T3	
n/N		533/1,540	85/223	94/258	99/241	
Median		0.0	0.2	0.6	1.2	
Model 1	1.03 (0.93–1.13)	1	1.03 (0.85–1.24)	0.98 (0.81–1.18)	1.10 (0.92–1.31)	0.39
Model 2	1.03 (0.93–1.13)	1	1.03 (0.85–1.26)	0.99 (0.82–1.19)	1.09 (0.91–1.30)	0.40
Model 3	1.04 (0.94–1.16)	1	1.03 (0.85–1.25)	0.99 (0.82–1.19)	1.12 (0.93–1.34)	0.30
Model 4	1.03 (0.93–1.14)	1	1.01 (0.83–1.22)	0.97 (0.80–1.16)	1.09 (0.91–1.30)	0.45
Cream		Zero	T1	T2	T3	
n/N		341/938	172/421	151/466	147/437	
Median		0.0	0.3	0.4	1.4	
Model 1	0.98 (0.96–1.01)	1	1.01 (0.86–1.20)	0.88 (0.75–1.03)	0.88 (0.74–1.04)	0.09
Model 2	0.98 (0.96–1.01)	1	1.03 (0.87–1.22)	0.89 (0.76–1.05)	0.87 (0.73–1.03)	0.06
Model 3	0.98 (0.96–1.01)	1	1.02 (0.86–1.21)	0.89 (0.76–1.05)	0.87 (0.74–1.03)	0.08
Model 4	0.98 (0.96–1.01)	1	1.03 (0.87–1.21)	0.90 (0.77–1.05)	0.87 (0.73–1.03)	0.06
Ice cream		Zero	T1	T2	T3	
n/N		263/718	98/280	287/783	163/481	
Median		0.00	0.02	0.05	0.13	
Model 1	0.82 (0.44–1.52)	1	1.04 (0.86–1.27)	0.99 (0.87–1.13)	0.96 (0.82–1.12)	0.49
Model 2	0.79 (0.43–1.48)	1	1.05 (0.86–1.27)	0.99 (0.87–1.14)	0.95 (0.81–1.12)	0.47
Model 3	0.79 (0.42–1.48)	1	1.06 (0.87–1.28)	0.99 (0.86–1.13)	0.95 (0.81–1.12)	0.44
Model 4	0.72 (0.38–1.38)	1	1.04 (0.86–1.26)	0.98 (0.86–1.12)	0.91 (0.78–1.08)	0.22

Model 1 included age (continuous), sex, follow-up duration and cohort. Model 2 was additionally adjusted for energy intake (continuous), education (3 categories), smoking (3 categories), physical activity (continuous), alcohol consumption (4 categories) and family history of diabetes (yes/no). Model 3 was additionally adjusted for food groups associated with type 2 diabetes including intakes of fruit, vegetables, tea, coffee, grains (whole and refined), meat (processed and red) and sugar-sweetened beverages (SSB) (continuous). Model 4 was additionally adjusted for BMI (continuous), LDL cholesterol (continuous), systolic and diastolic blood pressure (continuous). Significant associations are indicated in Bold

*HS* Hoorn Study, *T* Tertile, *Q* quartile, *RR* risk ratio

^1^Continuous analyses in servings/day: milk, 200 mL; yogurt, 150 mL; cheese, 20 g; cream, 3 g; ice cream, 100 g. Combined total dairy category: liquid dairy products, 200 mL; cheese, 20 g

Interactions were present between the exposures low fat dairy (*p* = 0.03), low fat fermented dairy (*p* = 0.01) and low fat cheese (*p* =  < 0.0001), and enrolment wave. In stratified analysis, low fat fermented dairy and low fat cheese were associated with prediabetes in HS1 (RR_servings/day_, respectively, 1.10, 95% CI 1.03–1.19, *p* = 0.01 and 1.33, 95% CI 1.20–1.47, *p* < 0.001) but not in HS2 (RR_servings/day_ 0.92, 95% CI 0.81–1.05 and 0.92, 95% CI 0.81–1.05) (Supplemental Table 3). Low fat cheese intake was much lower in HS1 as compared to HS2 (10.3% vs 46.2% of low fat fermented dairy). Furthermore, interactions with age were present for low fat dairy (*p* = 0.004), low fat fermented dairy (*p* < 0.001), yogurt (*p* = 0.01), low fat yogurt (*p* = 0.003), low fat cheese (*p* = 0.002) and interactions were present with BMI for low fat fermented dairy (*p* = 0.01) (Supplemental Table 4). Associations between these dairy exposures and prediabetes in participants aged 56 years and over, and in participants with a BMI ≥ 30 were similar as in HS1. Other stratified analysis were not significant.

None of the adjusted associations for substitution of high fat cheese for alternative dairy products were significant (Table 4). We further examined potential confounding of inverse associations between high fat cheese and prediabetes by assessing lifestyle and risk factors according to high fat cheese intake (Supplemental Table 5). In the highest compared to lowest quartile of high fat cheese intake, participants were more often male (53% vs 44%), current smoker (24% vs 17%), used less medication (antihypertensive 10% vs 17%; lipid lowering 2% vs 9%) and had higher LDL cholesterol levels (4.1 ± 1.1 vs 3.5 ± 1.0 mmol/L). Low fat cheese intake was lower and intakes of all other dairy types, energy and processed meat were higher.

**Table 4:** Risk ratio's (95% confidence interval) for the substitution of high fat cheese with alternative dairy products and incidence of prediabetes the Hoorn Studies (*n* = 2262)

High fat cheese	RR (95%CI)^1^
High fat milk, all types	1.06 (0.93–1.21)
Low fat milk, all types	1.06 (0.96–1.16)
High fat yogurt	1.20 (0.99–1.46)
Low fat yogurt	1.09 (0.95–1.26)
Low fat cheese	1.09 (0.98–1.22)
Cream	1.05 (0.98–1.12)
Ice cream	0.82 (0.44–1.54)

Substitution models included total servings/day of dairy intake and energy intake (kcal). Models were adjusted for age (continuous), sex, follow-up duration, cohort, education (3 categories), smoking (3 categories), physical activity (continuous), alcohol consumption (4 categories), family history of diabetes (yes/no), intakes of fruit, vegetables, tea, coffee, grains (whole and refined), meat (processed and red) and sugar-sweetened beverages (continuous). Significant associations are indicated in Bold

*HS* Hoorn Study, *RR* risk ratio

^1^Continuous analyses in servings/day: milk, 200 mL; yogurt, 150 mL; cheese, 20 g; cream, 3 g; ice cream, 100 g

### Sensitivity analysis

Mutual adjustment for intake of all other dairy types did not result in different associations (Supplementary Table 6). In the sample without CVD at baseline, associations between high fat fermented, total cheese and high fat cheese and prediabetes had similar effect estimates compared to associations in all participants, but these were no longer significant. Using Goldberg cut-offs, we identified 481 ‘under reporters’ (21%) and 62 ‘over reporters’ (3%). Repeating analyses in 1,716 ‘normal reporters’ (76%) resulted in stronger associations for high fat fermented dairy (RR_Q4vsQ1_ 0.79, 95% CI 0.65–0.97, *p*_trend_ = 0.04), total cheese (RR_Q4vsQ1_ 0.78, 95% CI 0.64–0.96, *p*_trend_ = 0.01, RR_servings/day_ 0.92, 95% CI 0.86–0.99) and high fat cheese (RR_Q4vsQ1_ 0.73, 95% CI 0.60–0.90, *p*_trend_ = 0.003, RR_servings/day_ 0.92, 95% CI 0.86–0.99). In ‘normal reporters’, continuous associations were significant for total dairy (RR_servings/day_ 0.95, 95% CI 0.91–0.99, *p* = 0.03) and total fermented dairy (RR_servings/day_ 0.95, 95% CI 0.90–1.00, *p* = 0.04) and prediabetes, although associations in quartiles were not. In analysis including participants with prediabetes at baseline, only high fat cheese remained significantly associated with prediabetes (RR_Q4vsQ1_ 0.85, 95% CI 0.73–0.98). Associations between high fat fermented and total cheese and prediabetes were attenuated but remained in the same direction. Sensitivity analyses of substitution of one serving/day of high fat cheese with alternative dairy types resulted in similar associations as in main analysis (Supplemental Table 7).

## Discussion

A high intake of high fat fermented dairy, total cheese and high fat cheese were associated with lower risk of prediabetes in this population-based cohort. Associations were driven by high fat cheese intake, as 52% and 83% of, respectively, high fat fermented dairy and total cheese intake consisted of high fat cheese. We found no associations for substitutions of high fat cheese with other dairy products and risk of prediabetes. Total dairy and milk, yogurt, cream and ice cream intake were not associated with prediabetes, irrespective of fat content.

Our observed associations of high fat cheese with prediabetes were consistent with the prospective FHS Offspring Cohort [9]. They reported a non-significant association for the top vs bottom intake of cheese intake (HR 0.86, 95% CI 0.69–1.07), although their cheese intake was considerably lower in the highest intake category compared to ours (median < 1 vs 2.6 servings/day). The cross-sectional Dutch Maastricht study reported an association for higher cheese intake with lower prediabetes risk (OR_20g/day_ 0.88, 95% CI 0.80–0.97) [13]. Similar inverse associations of cheese and 2hPG were also found in the longitudinal Inter99 study (β_20g/day_ − 0.05, 95% CI − 0.01, − 0.001 mmol/L) [11] and in the cross-sectional ELSA-Brasil (β_30/day_ − 0.05, 95% CI − 0.09, − 0.02 mmol/L) [15]. Our findings are in line with a review of three meta-analyses reporting moderate evidence of a prospective association between higher cheese intake and lower T2D risk [8], and the prospective Urban Rural Epidemiology (PURE) study including 131,481 individuals from 21 countries showing a 24% lower risk at > 1 servings/day compared to 0 servings/day (HR 0.76, 95% CI 0.64–0.91, *p*_trend_ = 0.001) [31]. Overall, current evidence indicates that higher cheese consumption is associated with lower prediabetes and type 2 diabetes risk.

Our results of no associations between total, low fat and high fat dairy and prediabetes are not in accordance with results from the FHS Offspring Cohort, which reported a lower risk of, respectively, 39%, 32% and 25% in the highest compared to lowest intake category [9]. These associations with prediabetes risk were driven by moderate consumption of low fat, skim milk and whole milk consumption, none of which were significant in our study. Several explanations may underscore these different findings, including a longer follow-up in their study (12 years vs 6.4 years), a higher prediabetes incidence (48.3% vs 35.9%), and a different prediabetes definition (no use of 2hPG and HbA1c, lower cut-off of 5.6 vs 6.0 mmol/L for FPG). Furthermore, they used repeated measurements of dairy intake during follow-up to account for within-person variability, which may have strengthened the associations. Also, in US populations, high dairy consumption is associated with an overall healthier dietary pattern [32], whereas in Europe, dairy consumption is more widespread across a range of populations.

Our results pointed towards effect modification of associations of low fat dairy types and yogurt with prediabetes by enrolment wave, age and BMI. Positive associations for low fat dairy types and prediabetes were shown in HS1, but not in HS2, which could be explained by differences in low fat dairy consumption patterns, such as negligible low fat cheese consumption in HS1. Changes in dairy intake observed between the two enrolment waves correspond to the changes observed from 1987/88 to 2007/10 as assessed in the Dutch National Food Consumption Survey [33]. They showed an increase in intake of dairy, especially of low fat types, skimmed and semi-skimmed yogurt and cheese. Furthermore, differences in characteristics between the enrolment waves could explain effect modification, especially as the HS1 population was somewhat older compared to HS2. Effect modification by enrolment wave may explain positive associations of low fat dairy types and prediabetes present in those with higher age and BMI, as these are not in line with previous studies [9, 34].

We found no associations of yogurt consumption with prediabetes, yet especially higher yogurt consumption has been associated to lower T2D risk in previous high quality research [8]. Significant nonlinear associations of yogurt with prediabetes were found in the FHS Offspring Cohort, with 25% risk reduction observed for 1 to ≤ 3 servings/week compared to no consumption, yet risk increased with higher intakes [9]. Inverse associations between yogurt and prediabetes were also reported by two cross-sectional studies [13, 15], but no associations were found in the much larger Lifelines study [14]. Our neutral observations for yogurt could be due to the inclusion of porridge and custard in this category for HS2, as the HS2 FFQ combined these in a single question. Furthermore, yogurt consumption has been related to healthier diet and lifestyle [13, 35], and residual confounding could explain discrepancies in results across studies.

There are several mechanisms that may explain the observed associations of cheese with prediabetes. Despite an average fat content of 24–35 g/100 g [36] of which 70% SFAs, various RCTs demonstrated less adverse effects of SFAs contained within the cheese matrix compared to SFAs in different matrixes [36–38]. Beneficial associations have been found between ruminant trans fatty acids and insulin resistance and type 2 diabetes [39, 40], with potential mechanisms suggested in animal studies being inhibition of hepatic de novo lipogenesis, activation of PPAR-α and PPAR-γ, improving insulin sensitivity and reducing inflammation [40, 41]. As shown by meta-analysis of 15 RCTs [42], calcium may affect energy balance by increasing faecal fat excretion, due to formation of insoluble Ca-fatty acid soaps and/or formation of hydrophobic aggregations. However, in a meta-analysis of 20 RCTs, calcium supplementation did not reduce body weight or body fat [43]. Fermented foods contain lactic acid bacteria and bioactive molecules, which are beneficial for viability and composition of the gut microbiota and influence gene expression related to glucose and insulin metabolism [41, 44]. Furthermore, vitamin K2 (menaquinones) in dairy is synthesized during fermentation, and cheese is the richest source of vitamin K2 in Western diets (12.7 μg/20 g) [45]. Higher vitamin K2 intake has been related to lower T2D risk in the Dutch EPIC cohort [46]. In animal models of T2D, vitamin K2 supplementation showed dose-dependent reductions of HbA1c and FPG and improved insulin resistance and β-cell function [47, 48]. Vitamin K2 may upregulate carboxylated osteocalcin, resulting in increased serum adiponectin levels, which enhances insulin sensitivity through increased fatty acid oxidation in skeletal muscles and inhibition of hepatic glucose production in the liver [49]. Whether the vitamin K2 induced pathways underline long-term effects of cheese warrants further investigation.

Despite multiple studies pointing to a role of cheese in diabetes prevention, the exact place of cheese in healthy diets is unclear. Current American and European dietary guidelines only advice low fat cheese to limit intake of SFAs and sodium [50], although evidence of more favourable associations of low fat cheese with cardiometabolic outcomes is lacking [51]. The limited evidence available is not sufficient to justify changes to dietary guidelines, and additional well-designed controlled trials are needed.

This study has several strengths, including the assessment of a wide range of dairy subtypes and possibility to disseminate low and high fat types, also for yogurt and cheese. Other strengths include the longitudinal design with 6 years of follow-up and extensive adjustments for confounders. Despite these notable strengths, there are certain limitations that should be mentioned. First, although both FFQs were validated [18, 19], measurement errors in reported dietary intake due to recall bias are unavoidable. We corrected for energy misreporting and found slightly stronger associations in ‘normal reporters’, indicating attenuated effect sizes in main analyses due to energy misreporting. The FFQs used in the two waves were overall comparable, and slight differences in brands and dairy products included largely reflect the changes in dietary patterns between two time periods [33], for example the inclusion of more low fat cheese types in the HS2 cohorts. No compositional changes in dairy products were observed between both NEVO-tables used for calculating nutrient intakes by the FFQ. The main findings and conclusions were similar when stratifying associations by enrolment wave. Second, no repeated measurements of diet were available, and although dietary patterns have shown to be somewhat stable [52, 53], for dairy product intake specifically in comparison to other food groups [53], errors in single dietary measurements may result in bias of associations towards the null. We addressed reverse causality due to dietary changes related to diagnoses of disease by excluding participants with history of diabetes, and excluding participants with prevalent CVD, resulting in similar associations although no longer significant, likely as a result of less power. Lastly, identification of prediabetes cases was less sensitive in HS2 compared to HS1, because no OGTT was done at follow-up and capillary sample HbA1c levels were used, which tend to be higher than venous sample HbA1c levels [54]. Furthermore, reproducibility of FPG and 2hPG is only moderate [55], and participants may revert to normoglycemia during follow-up [5]. We addressed this possible misclassification by including participants with prediabetes at baseline in sensitivity analysis, showing similar significant associations for high fat cheese in quartiles, and other associations remained in similar direction.

In conclusion, higher consumption of high fat cheese was continuously associated with lower risk of prediabetes, suggesting a potential preventive role of cheese in T2D development. Observed associations between higher high fat fermented diary and total cheese consumption and lower prediabetes risk were likely driven by high fat cheese consumption. We found no associations between total dairy, fermented dairy, milk, yogurt, cream and ice cream, regardless of fat content, with prediabetes development. Further prospective and intervention research is needed to elucidate health effects of cheese considering high SFAs and sodium content, and its place in healthy diets.

## Supplementary Information

Below is the link to the electronic supplementary material.

Supplementary file1 (DOCX 126 KB)

## Data Availability

Data described in the manuscript will be made available upon request pending approval by the authors and approval of the request form, which can be found at https://hoornstudies.com/cohorts-data-request.php.

## Code availability

Analytic code (R syntax) will be made available upon request pending approval by the authors.
