# Supplementary material for: Dairy product consumption and incident prediabetes in Dutch middle-aged adults: the Hoorn Studies prospective cohort
Source: Eur J Nutr. 2021 Jul 10;61(1):183–96. doi: 10.1007/s00394-021-02626-9 (PMC8783852; doi:10.1007/s00394-021-02626-9)
Supplement: Supplementary file 1 — Supplementary file1 (DOCX 126 KB) [file 394_2021_2626_MOESM1_ESM.docx]

# Supplemental figures and tables

**Supplemental Figure 1.** *Flow chart of the study population. HS; Hoorn Study.*

| **Supplemental Table 1.** *Baseline characteristics and dairy intakes of participants of the Hoorn Studies according to enrolment wave (n=2,262)* | | |  |
| --- | --- | --- | --- |
|  | **Enrolment wave** | | |
|  | HS1 (*n*=997) | HS2 (*n*=1265) | |
| Follow-up time (y) | 6.0 ± 0.4 | 6.7 ± 0.7 | |
| Sex (men) | 56% (563) | 45% (569) | |
| Age (y) | 59.6 ± 6.6 | 53.0 ± 6.6 | |
| Education level |  |  | |
| Low | 25% (251) | 4% (48) | |
| Middle | 62% (619) | 55% (691) | |
| High | 13% (126) | 40% (505) | |
| Smoking |  |  | |
| Current | 28% (282) | 17% (211) | |
| Previous (>2 months ago) | 35% (346) | 40% (505) | |
| Never | 37% (368) | 43% (540) | |
| Cigarette years | 160 (0-460) | 240 (100-510) | |
| Alcohol intake |  |  | |
| 0 g/day | 25% (250) | 12% (151) | |
| ≤10 g/day | 43% (432) | 42% (527) | |
| 10-30 g/day | 23% (232) | 35% (446) | |
| ≥30 g/day | 8% (83) | 11% (140) | |
| Physical activity, moderate intensity, hours/week | 9.0 (5.3-14.0) | 6.5 (3.5-10.5) | |
| Family history diabetes mellitus | 24% (243) | 25% (310) | |
| BMI (kg/m^2^) | 25.9 ± 3.0 | 25.6 ± 3.6 | |
| Fasting glucose (mmol/L) | 5.2 ± 0.4 | 5.3 ± 0.4 | |
| Systolic blood pressure (mmHg) | 130 ± 19 | 130 ± 16 | |
| Diastolic blood pressure (mmHg) | 81 ± 10 | 76 ± 10 | |
| Antihypertensive medication use | 12% (122) | 14% (182) | |
| LDL cholesterol (mmol/L) | 1.4 ± 0.8 | 3.3 ± 0.9 | |
| Lipid lowering medication | 1% (11) | 8% (97) | |
| **Dietary intake** |  |  | |
| Energy intake (kcal/day) | 2100 ± 540 | 2200 ± 630 | |
| DHD15-index score | 71 ± 13 | 70 ± 14 | |
| Fruit (g/day) | 240 ± 150 | 160 ± 120 | |
| Vegetables (g/day) | 110 ± 46 | 190 ± 92 | |
| Grain (g/day) | 150 ± 65 | 230 ± 100 | |
| Red meat (g/day) | 27 ± 22 | 39 ± 22 | |
| Processed meat (g/day) | 61 ± 37 | 33 ± 22 | |
| Lean fish (g/day) | 11 ± 14 | 11 ± 11 | |
| Fatty fish (g/day) | 4.8 ± 9.7 | 5.1 ± 7.3 | |
| Coffee (g/day) | 550 ± 270 | 460 ± 260 | |
| Tea (g/day) | 310 ± 270 | 260 ± 260 | |
| Fruit juice (g/day) | 33 ± 85 | 81 ± 97 | |
| Sugar-sweetened beverages (g/day) | 83 ± 130 | 140 ± 150 | |
| Saturated fat (en%) | 17.0 ± 3.3 | 12.7 ± 2.8 | |
| Protein (en%) | 14.7 ± 2.6 | 14.4 ± 2.2 | |
| Calcium (g/day) | 1100 ± 390 | 960 ± 330 | |
| **Dairy intake (servings/day)** |  |  | |
| Total dairy | 3.7 ± 1.7 | 2.5 ± 1.4 | |
| High fat dairy | 2.1 ± 1.4 | 1.1 ± 1.2 | |
| Low fat dairy | 1.6 ± 1.4 | 1.3 ± 1.0 | |
| Milk, all types | 1.5 ± 1.2 | 0.8 ± 0.7 | |
| High fat milk, all types | 0.3 ± 0.6 | 0.1 ± 0.3 | |
| Low fat milk, all types | 1.1 ± 1.2 | 0.7 ± 0.6 | |
| Milk, regular | 1.3 ± 1.2 | 0.6 ± 0.6 | |
| High fat milk, regular | 0.2 ± 0.6 | 0.04 ± 0.24 | |
| Low fat milk, regular | 1.1 ± 1.2 | 0.6 ± 0.6 | |
| Fermented dairy | 2.7 ± 1.5 | 1.8 ± 1.2 | |
| High fat fermented dairy | 1.6 ± 1.2 | 1.0 ± 1.1 | |
| Low fat fermented dairy | 1.1 ± 1.1 | 0.8 ± 0.7 | |
| Yogurt | 0.6 ± 0.6 | 0.5 ± 0.4 | |
| High fat yogurt | 0.2 ± 0.4 | 0.1 ± 0.3 | |
| Low fat yogurt | 0.4 ± 0.6 | 0.4 ± 0.4 | |
| Cheese | 1.5 ± 1.1 | 1.3 ± 1.1 | |
| High fat cheese | 1.4 ± 1.1 | 0.9 ± 1.1 | |
| Low fat cheese | 0.1 ± 0.4 | 0.4 ± 0.6 | |
| Cream | 0.5 ± 2.0 | 1.1 ± 2.8 | |
| Ice cream | 0.1 ± 0.1 | 0.1 ± 0.1 | |
| Variables are displayed as means ± SD for normally distributed continuous variables, medians (IQR) for non-normally distributed continuous variables or % (n) for categorical variables.  HS1; Hoorn Studies 1 (first enrolment wave), HS2; Hoorn Studies 2 (second enrolment wave) | | |  |

| **Supplementary Table 2.** *Baseline characteristics of participants of the Hoorn Studies according to whether participants had complete follow-up or were lost-to-follow-up.* | | |
| --- | --- | --- |
|  | Complete follow-up (*n*=3,245) | Lost-to-follow-up (*n*=2,046) |
| Sex (men) | 51% (1,645) | 49% (1,008) |
| Age (y) | 56.9 ± 7.6 | 58.0 ± 9.0 |
| Education level |  |  |
| Low | 15% (494) | 22% (444) |
| Middle | 58% (1,857) | 57% (1,146) |
| High | 27% (849) | 20% (408) |
| Smoking |  |  |
| Current | 23% (748) | 31% (617) |
| Previous (>2 months ago) | 39% (1,252) | 35% (696) |
| Never | 38% (1,223) | 35% (702) |
| Alcohol intake g/day | 7.2 (2.0-17.2) | 5.0 (0.0-15.3) |
| Physical activity, moderate intensity, hours/week | 7.5 (4.0-12.3) | 7.0 (3.5-12.7) |
| BMI (kg/m^2^) | 26.3 ± 3.6 | 26.6 ± 4.1 |
| Fasting glucose (mmol/L) | 5.6 ± 1.0 | 5.8 ± 1.6 |
| Variables are displayed as means ± SD for normally distributed continuous variables, medians (IQR) for non-normally distributed continuous variables or % (n) for categorical variables. | | |

| **Supplemental table 3.** *Risk ratio's (95% confidence interval) for the association between dairy intake and incidence of prediabetes the Hoorn Studies, stratified for the first enrolment wave in 1989-1992 (Hoorn Study 1, HS1, n=997) and a second wave in 2006-2007 (HS2, n=1,265)* | | | | | | |
| --- | --- | --- | --- | --- | --- | --- |
|  | **Continuous^1^** | **Intake range categories** | | | |  |
|  | RR (95%CI) |  | RR (95%CI) | RR (95%CI) | RR (95%CI) | P_trend_ |
| **Total dairy** |  | **Q1** | **Q2** | **Q3** | **Q4** |  |
| HS1: median; n/N |  | 1.9; 77/249 | 2.9; 83/248 | 3.8; 87/249 | 5.5; 82/251 |  |
| HS1: model 3 | 1.00 (0.94-1.06) | 1 | 1.11 (0.86-1.44) | 1.15 (0.89-1.48) | 1.02 (0.77-1.35) | 0.98 |
| HS2: median; n/N |  | 1.2; 122/317 | 1.8; 124/316 | 2.5; 123/314 | 3.5; 113/318 |  |
| HS2: model 3 | 0.96 (0.91-1.02) | 1 | 1.01 (0.83-1.23) | 1.01 (0.83-1.23) | 0.92 (0.74-1.14) | 0.41 |
| **High fat dairy** |  | **Q1** | **Q2** | **Q3** | **Q4** |  |
| HS1: median; n/N |  | 0.9; 89/243 | 1.7; 82/254 | 2.0; 71/249 | 3.2; 87/251 |  |
| HS1: model 3 | 0.92 (0.85-1.00) | 1 | 0.86 (0.68-1.10) | 0.78 (0.59-1.01) | 0.88 (0.67-1.14) | 0.60 |
| HS2: median; n/N |  | 0.0; 114/315 | 0.7; 122/318 | 1.4; 127/314 | 2.0; 119/318 |  |
| HS2: model 3 | 0.99 (0.92-1.06) | 1 | 1.05 (0.86-1.29) | 1.16 (0.94-1.42) | 1.09 (0.87-1.37) | 0.53 |
| **Low fat dairy** |  | **Q1** | **Q2** | **Q3** | **Q4** |  |
| HS1: median; n/N |  | 0.0; 75/245 | 1.0; 91/250 | 1.8; 86/250 | 3.1; 77/252 |  |
| HS1: model 3 | 1.06 (0.99-1.13) | 1 | 1.22 (0.95-1.57) | 1.16 (0.90-1.50) | 1.01 (0.77-1.33) | 0.84 |
| HS2: median; n/N |  | 0.3; 116/309 | 0.8; 128/325 | 1.4; 124/317 | 2.2; 114/314 |  |
| HS2: model 3 | 0.95 (0.88-1.02) | 1 | 1.03 (0.84-1.25) | 1.03 (0.84-1.25) | 0.96 (0.77-1.18) | 0.64 |
| **Total fermented dairy** |  | **Q1** | **Q2** | **Q3** | **Q4** |  |
| HS1: median; n/N |  | 1.1; 80/247 | 2.0; 69/249 | 2.8; 92/251 | 4.1; 88/250 |  |
| HS1: model 3 | 1.02 (0.95-1.08) | 1 | 0.87 (0.66-1.14) | 1.16 (0.90-1.49) | 1.13 (0.87-1.47) | 0.14 |
| HS2: median; n/N |  | 0.7; 125/326 | 1.3; 122/308 | 1.7; 121/315 | 2.7; 114/316 |  |
| HS2: model 3 | 0.96 (0.89-1.02) | 1 | 1.05 (0.86-1.27) | 0.99 (0.81-1.21) | 0.95 (0.77-1.16) | 0.51 |
| **High fat fermented dairy** |  | **Q1** | **Q2** | **Q3** | **Q4** |  |
| HS1: median; n/N |  | 0.3; 84/235 | 1.1; 84/258 | 2.1; 79/253 | 2.6; 82/251 |  |
| HS1: model 3 | 0.92 (0.84-1.01) | 1 | 0.91 (0.71-1.16) | 0.91 (0.70-1.17) | 0.93 (0.72-1.21) | 0.82 |
| HS2: median; n/N |  | 0.0; 124/316 | 0.5; 120/317 | 1.3; 121/316 | 2.0; 117/316 |  |
| HS2: model 3 | 0.98 (0.91-1.05) | 1 | 0.95 (0.78-1.16) | 1.00 (0.82-1.22) | 0.98 (0.79-1.22) | 0.99 |
| **Low fat fermented dairy** |  |  |  |  |  |  |
| HS1: median; n/N |  | **Z:** 0.0; 152/498 | **T1:** 0.3; 25/72 | **T2:** 1.0; 119/349 | **T3:** 2.2; 33/78 |  |
| HS1: model 3 | 1.10 (1.03-1.19) | 1 | 0.86 (0.66-1.13) | 0.99 (0.76-1.29) | 1.02 (0.78-1.33) | 0.39 |
| HS2: median; n/N |  | **Q1:** 0.0; 123/315 | **Q2:** 0.4; 127/318 | **Q3:** 0.7; 113/319 | **Q4:** 1.3; 119/313 |  |
| HS2: model 3 | 0.93 (0.83-1.03) | 1 | 1.06 (0.87-1.30) | 0.94 (0.76-1.16) | 0.96 (0.79-1.18) | 0.48 |
| **Total milk, all types** |  | **Q1** | **Q2** | **Q3** | **Q4** |  |
| HS1: median; n/N |  | 0.2; 71/244 | 0.9; 96/252 | 1.6; 76/251 | 2.8; 86/250 |  |
| HS1: model 3 | 1.00 (0.92-1.08) | 1 | 1.31 (1.02-1.67) | 1.05 (0.80-1.37) | 1.10 (0.84-1.44) | 1.00 |
| HS2: median; n/N |  | 0.0; 122/320 | 0.5; 115/312 | 0.8; 126/317 | 1.5; 119/316 |  |
| HS2: model 3 | 1.00 (0.89-1.11) | 1 | 0.98 (0.80-1.20) | 1.03 (0.84-1.25) | 0.98 (0.80-1.20) | 0.91 |
| **High fat milk, all types** |  | **Zero** | **T1** | **T2** | **T3** |  |
| HS1: median; n/N |  | 0.0; 113/360 | 0.1; 58/164 | 0.2; 79/260 | 0.9; 79/213 |  |
| HS1: model 3 | 0.95 (0.82-1.11) | 1 | 1.17 (0.89-1.53) | 0.96 (0.75-1.23) | 1.11 (0.87-1.43) | 0.45 |
| HS2: median; n/N |  | 0.0; 384/979 | 0.01; 30/95 | 0.04; 23/94 | 0.3; 45/97 |  |
| HS2: model 3 | 1.14 (0.93-1.41) | 1 | 0.88 (0.64-1.19) | 0.64 (0.44-0.93) | 1.17 (0.92-1.49) | 0.19 |
| **Low fat milk, all types** |  | **Q1** | **Q2** | **Q3** | **Q4** |  |
| HS1: median; n/N |  | 0.0; 82/248 | 0.5; 76/246 | 1.3; 89/252 | 2.5; 82/251 |  |
| HS1: model 3 | 1.01 (0.93-1.09) | 1 | 0.96 (0.74-1.23) | 1.09 (0.85-1.39) | 0.98 (0.76-1.27) | 0.93 |
| HS2: median; n/N |  | 0.0; 121/319 | 0.4; 115/314 | 0.8; 130/315 | 1.4; 116/317 |  |
| HS2: model 3 | 0.97 (0.86-1.09) | 1 | 0.99 (0.81-1.21) | 1.09 (0.89-1.32) | 0.98 (0.80-1.20) | 0.94 |
| **Total regular milk** |  | **Q1** | **Q2** | **Q3** | **Q4** |  |
| HS1: median; n/N |  | 0.0; 70/249 | 0.8; 98/248 | 1.4; 75/246 | 2.6; 86/254 |  |
| HS1: model 3 | 1.00 (0.93-1.08) | 1 | 1.39 (1.09-1.78) | 1.07 (0.82-1.41) | 1.12 (0.85-1.46) | 0.98 |
| HS2: median; n/N |  | 0.0; 123/326 | 0.3; 90/251 | 0.7; 161/403 | 1.4; 108/285 |  |
| HS2: model 3 | 1.01 (0.90-1.13) | 1 | 0.96 (0.77-1.20) | 1.04 (0.86-1.25) | 0.99 (0.80-1.22) | 0.93 |
| **High fat regular milk** |  | **Zero** | **T1** | **T2** | **T3** |  |
| HS1: median; n/N |  | 0.0; 259/795 | 0.3; 19/62 | 0.8; 32/72 | 2.0; 19/68 |  |
| HS1: model 3 | 0.94 (0.80-1.10) | 1 | 0.88 (0.60-1.30) | 1.31 (0.98-1.75) | 0.76 (0.51-1.13) | 0.41 |
| HS2: median; n/N |  | 0.0; 452/1,194 | 0.2; 8/24 | 0.3; 8/18 | 1.4; 14/29 |  |
| HS2: model 3 | 1.14 (0.91-1.41) | 1 | 0.91 (0.51-1.62) | 1.17 (0.69-1.96) | 1.16 (0.78-1.73) | 0.44 |
| **Low fat regular milk** |  |  |  |  |  |  |
| HS1: median; n/N |  | **Q1**: 0.0; 82/249 | **Q2:** 0.4; 76/246 | **Q3:** 1.1; 88/249 | **Q4:** 2.5; 83/253 |  |
| HS1: model 3 | 1.02 (0.94-1.10) | 1 | 0.96 (0.75-1.24) | 1.09 (0.85-1.39) | 0.99 (0.77-1.28) | 0.93 |
| HS2: median; n/N |  | **Z:** 0.0; 122/322 | **T1:** 0.2; 107/288 | **T2:** 0.7; 136/340 | **T3:** 1.4; 117/315 |  |
| HS2: model 3 | 0.98 (0.87-1.11) | 1 | 1.00 (0.81-1.23) | 1.04 (0.86-1.27) | 0.98 (0.80-1.21) | 0.92 |
| **Total yogurt** |  | **Q1** | **Q2** | **Q3** | **Q4** |  |
| HS1: median; n/N |  | 0.0; 74/248 | 0.2; 78/210 | 0.7; 77/284 | 1.2; 100/255 |  |
| HS1: model 3 | 1.13 (0.97-1.31) | 1 | 1.32 (1.02-1.70) | 0.96 (0.73-1.26) | 1.37 (1.07-1.76) | 0.10 |
| HS2: median; n/N |  | 0.0; 113/278 | 0.4; 126/349 | 0.6; 132/323 | 0.9; 111/315 |  |
| HS2: model 3 | 0.94 (0.79-1.12) | 1 | 0.91 (0.75-1.12) | 1.02 (0.83-1.25) | 0.88 (0.71-1.08) | 0.41 |
| **High fat yogurt** |  | **Zero** | **T1** | **T2** | **T3** |  |
| HS1: median; n/N |  | 0.0; 238/734 | 0.1; 31/86 | 0.6; 25/89 | 1.1; 35/88 |  |
| HS1: model 3 | 1.09 (0.87-1.37) | 1 | 1.14 (0.84-1.55) | 0.89 (0.62-1.27) | 1.18 (0.88-1.58) | 0.50 |
| HS2: median; n/N |  | 0.0; 312/841 | 0.1; 57/140 | 0.2; 62/160 | 0.6; 51/124 |  |
| HS2: model 3 | 1.05 (0.80-1.39) | 1 | 1.18 (0.95-1.47) | 1.11 (0.90-1.38) | 1.17 (0.92-1.49) | 0.18 |
| **Low fat yogurt** |  |  |  |  |  |  |
| HS1: median; n/N |  | **Z:** 0.0; 144/440 | **T1:** 0.2; 67/185 | **T2:** 0.7; 48/178 | **T3:** 1.1; 70/194 |  |
| HS1: model 3 | 1.09 (0.93-1.28) | 1 | 1.14 (0.90-1.44) | 0.84 (0.64-1.11) | 1.15 (0.91-1.45) | 0.67 |
| HS2: median; n/N |  | **Q1:** 0.0; 123/318 | **Q2:** 0.2; 159/418 | **Q3:** 0.5; 45/127 | **Q4:** 0.9; 155/402 |  |
| HS2: model 3 | 0.91 (0.75-1.10) | 1 | 1.02 (0.84-1.23) | 0.97 (0.73-1.29) | 1.00 (0.82-1.21) | 0.89 |
| **Total cheese** |  | **Q1** | **Q2** | **Q3** | **Q4** |  |
| HS1: median; n/N |  | 0.5; 79/239 | 1.0; 82/233 | 1.6; 89/269 | 2.6; 79/256 |  |
| HS1: model 3 | 0.96 (0.88-1.05) | 1 | 1.05 (0.82-1.35) | 0.96 (0.75-1.23) | 0.97 (0.75-1.27) | 0.70 |
| HS2: median; n/N |  | 0.2; 128/316 | 0.7; 131/315 | 1.4; 125/360 | 2.7; 98/274 |  |
| HS2: model 3 | 0.96 (0.89-1.03) | 1 | 1.02 (0.84-1.23) | 0.85 (0.70-1.03) | 0.88 (0.71-1.10) | 0.14 |
| **High fat cheese** |  | **Q1** | **Q2** | **Q3** | **Q4** |  |
| HS1: median; n/N |  | 0.2; 87/239 | 1.0; 82/239 | 1.5; 79/216 | 2.3; 81/303 |  |
| HS1: model 3 | 0.89 (0.81-0.99) | 1 | 0.90 (0.71-1.15) | 0.95 (0.74-1.21) | 0.73 (0.56-0.95) | 0.02 |
| HS2: median; n/N |  | 0.0; 128/317 | 0.4; 117/311 | 0.9; 127/320 | 1.9; 110/317 |  |
| HS2: model 3 | 0.98 (0.90-1.05) | 1 | 0.92 (0.76-1.13) | 0.99 (0.81-1.20) | 0.86 (0.69-1.07) | 0.25 |
| **Low fat cheese** |  | **Zero** | **T1** | **T2** | **T3** |  |
| HS1: median; n/N |  | 0.0; 292/919 | 0.5; 10/26 | 1.0; 11/26 | 2.0; 16/26 |  |
| HS1: model 3 | 1.33 (1.20-1.47) | 1 | 1.34 (0.85-2.11) | 1.36 (0.84-2.21) | 1.93 (1.41-2.64) | 0.00002 |
| HS2: median; n/N |  | 0.0; 241/621 | 0.2; 82/216 | 0.5; 77/214 | 1.1; 82/214 |  |
| HS2: model 3 | 0.92 (0.81-1.05) | 1 | 0.98 (0.80-1.20) | 0.91 (0.74-1.12) | 0.98 (0.80-1.20) | 0.74 |
| **Cream** |  |  |  |  |  |  |
| HS1: median; n/N |  | **Z:** 0.0; 230/660 | **M1:** 0.3; 66/220 | **M2:** 1.3; 33/117 | NA |  |
| HS1: model 3 | 0.98 (0.92-1.04) | 1 | 1.00 (0.79-1.27) | 0.95 (0.77-1.17) | NA | 0.93 |
| HS2: median; n/N |  | **Z:** 0.0; 111/278 | **T1:** 0.1; 88/223 | **T2:** 0.4; 175/448 | **T3:** 1.5; 108/316 |  |
| HS2: model 3 | 0.98 (0.96-1.01) | 1 | 0.99 (0.80-1.23) | 1.00 (0.83-1.20) | 0.87 (0.70-1.08) | 0.15 |
| **Ice cream** |  | **Zero** | **T1** | **T2** | **T3** |  |
| HS1: median; n/N |  | 0.0; 263/718 | 0.02; 98/280 | 0.1; 287/783 | 0.1; 163/481 |  |
| HS1: model 3 | 0.89 (0.28-2.81) | 1 | 1.05 (0.83-1.34) | 0.96 (0.75-1.24) | 0.94 (0.73-1.20) | 0.47 |
| HS2: median; n/N |  | 0.0; 263/718 | 0.02; 98/280 | 0.1; 287/783 | 0.1; 163/481 |  |
| HS2: model 3 | 0.76 (0.35-1.64) | 1 | 1.26 (0.88-1.81) | 0.98 (0.84-1.15) | 0.95 (0.77-1.19) | 0.58 |
| ^1^ Continuous analyses in servings/day: milk, 200 ml; yogurt, 150 ml; cheese, 20 g; cream 3 g; ice cream, 100 g. Combined total dairy category: liquid dairy products, 200 ml; cheese, 20g.  Model 3 included age (continuous), sex, follow-up duration, cohort, energy intake (continuous), education (3 categories), smoking (3 categories), physical activity (continuous), alcohol consumption (4 categories), family history of diabetes (yes/no), intakes of fruit, vegetables, tea, coffee, grains (whole and refined), meat (processed and red) and sugar-sweetened beverages (continuous). HS, Hoorn Study; Q, quartile; RR, Risk Ratio; T, Tertile; Z, Zero intake category. Significant associations are indicated in **Bold.** | | | | | | |

| **Supplemental Table 4.** *Risk ratio's (95% confidence interval) for stratified associations between dairy intake and incidence of prediabetes: analysis stratified by significant effect modifiers, the Hoorn Studies (n=2,262).* | | | | |
| --- | --- | --- | --- | --- |
| Dairy type | Strata | n/N | RR (95%CI)^1^ | *P*_interaction_ |
| *Interaction with age^2^* |  |  |  |  |
| Low fat dairy | Age <56 | 376/1131 | 0.94 (0.86-1.02) | 0.004 |
|  | Age ≥56 | 435/1131 | 1.06 (1.00-1.12) |  |
|  |  |  |  |  |
| Low fat fermented dairy | Age <56 | 376/1131 | 0.92 (0.83-1.03) | 0.0002 |
|  | Age ≥56 | 435/1131 | **1.10 (1.02-1.18)** |  |
|  |  |  |  |  |
| Yogurt | Age <56 | 376/1131 | 0.95 (0.79-1.15 | 0.01 |
|  | Age ≥56 | 435/1131 | 1.11 (0.96-1.28) |  |
|  |  |  |  |  |
| Low fat yogurt | Age <56 | 376/1131 | 0.82 (0.66-1.03) | 0.004 |
|  | Age ≥56 | 435/1131 | 1.14 (0.99-1.32) |  |
|  |  |  |  |  |
| Low fat cheese | Age <56 | 376/1131 | 0.88 (0.73-1.07) | 0.002 |
|  | Age ≥56 | 435/1131 | **1.15 (1.03-1.29)** |  |
|  |  |  |  |  |
| *Interaction with BMI^3^* |  |  |  |  |
| Low fat fermented dairy | BMI <25 | 313/1009 | 0.99 (0.89-1.10) | 0.01 |
|  | BMI 25-30 | 383/1010 | 1.01 (0.93-1.11) |  |
|  | BMI ≥30 | 113/239 | **1.14 (1.01-1.28)** |  |
| ^1^ Continuous analyses in servings/day: yogurt, 150 ml; cheese, 20 g.  ^2^ Models were adjusted for sex, follow-up duration, cohort, education (3 categories), smoking (3 categories), physical activity (continuous), alcohol consumption (4 categories), family history of diabetes (yes/no), intakes of fruit, vegetables, tea, coffee, grains (whole and refined), meat (processed and red) and sugar-sweetened beverages (continuous).  ^3^ Model adjusted additionally for age (continuous).  RR, Risk Ratio. Significant associations are indicated in **Bold.** | | | | |

| **Supplemental Table 5.** *Baseline characteristics and dietary intakes of participants of the Hoorn Studies according to quartiles of high fat cheese intake (n=2,262)* | | | | | | |
| --- | --- | --- | --- | --- | --- | --- |
|  | N missing | **Total population** | **Q1** | **Q2** | **Q3** | **Q4** |
|  |  | (n=2,262) | (n=558) | (n=600) | (n=532) | (n=572) |
| High fat cheese intake |  |  |  |  |  |  |
| Median |  | 0.9 ± 1.5 | 0.0 ± 0.1 | 0.6 ± 0.3 | 1.2 ± 0.4 | 2.3 ± 0.9 |
| Follow-up time (y) |  | 6.4 ± 0.70 | 6.5 ± 0.72 | 6.4 ± 0.71 | 6.3 ± 0.65 | 6.3 ± 0.70 |
| Sex (men) |  | 50% (1132) | 44% (246) | 48% (285) | 56% (297) | 53% (304) |
| Age (y) |  | 56 ± 7.3 | 55 ± 7.3 | 55 ± 7.3 | 57 ± 7.8 | 56 ± 6.8 |
| Education level | 22 |  |  |  |  |  |
| Low |  | 13% (299) | 11% (64) | 10% (63) | 18% (97) | 13% (75) |
| Middle |  | 58% (1310) | 59% (329) | 60% (363) | 55% (292) | 57% (326) |
| High |  | 28% (631) | 27% (152) | 29% (172) | 26% (139) | 29% (168) |
| Smoking | 10 |  |  |  |  |  |
| Current |  | 22% (493) | 17% (96) | 23% (137) | 23% (121) | 24% (139) |
| Previous (>2 months ago) |  | 38% (851) | 41% (231) | 33% (199) | 39% (210) | 37% (211) |
| Never |  | 40% (908) | 41% (228) | 43% (258) | 38% (201) | 39% (221) |
| Cigarette years | 654 | 210 (9-480) | 210 (64-470) | 230 (15-500) | 180 (0-480) | 200 (0-470) |
| Alcohol intake | 1 |  |  |  |  |  |
| 0 g/day |  | 18% (401) | 19% (106) | 19% (113) | 18% (97) | 15% (85) |
| ≤10 g/day |  | 42% (959) | 45% (251) | 41% (248) | 42% (225) | 41% (235) |
| 10-30 g/day |  | 30% (678) | 27% (149) | 32% (189) | 30% (159) | 32% (181) |
| ≥30 g/day |  | 10% (223) | 9% (52) | 8% (49) | 10% (51) | 12% (71) |
| Physical activity, moderate intensity, hours/week | 47 | 7.5 (4.2-12) | 6.8 (3.5-11) | 7.3 (3.8-12) | 7.5 (4.6-12) | 8.5 (5.0-13) |
| BMI (kg/m2) | 4 | 26 ± 3.4 | 26 ± 3.5 | 26 ± 3.2 | 26 ± 3.2 | 26 ± 3.5 |
| Fasting glucose (mmol/L) | 7 | 5.3 ± 0.39 | 5.3 ± 0.38 | 5.3 ± 0.37 | 5.2 ± 0.40 | 5.3 ± 0.40 |
| Systolic blood pressure (mmHg) | 2 | 130 ± 17 | 130 ± 17 | 130 ± 17 | 130 ± 19 | 130 ± 17 |
| Diastolic blood pressure (mmHg) | 5 | 78 ± 11 | 77 ± 10 | 79 ± 10 | 79 ± 11 | 79 ± 11 |
| Antihypertensive medication use |  | 13% (304) | 17% (96) | 15% (92) | 11% (61) | 10% (55) |
| LDL cholesterol mmol/L) | 4 | 3.8 ± 1.1 | 3.5 ± 1.0 | 3.7 ± 1.1 | 4.0 ± 1.1 | 4.1 ± 1.1 |
| Lipid lowering medication |  | 5% (108) | 9% (51) | 4% (27) | 3% (16) | 2% (14) |
| Family history diabetes mellitus | 11 | 24% (553) | 27% (153) | 26% (154) | 25% (133) | 20% (113) |
| **Dairy intake (servings/day)** |  |  |  |  |  |  |
| Total dairy |  | 3.0 ± 1.7 | 1.9 ± 1.3 | 2.4 ± 1.1 | 3.2 ± 1.3 | 4.6 ± 1.6 |
| High fat dairy |  | 1.5 ± 1.3 | 0.3 ± 0.5 | 1.0 ± 0.7 | 1.8 ± 0.8 | 3.1 ± 1.2 |
| Low fat dairy |  | 1.5 ± 1.2 | 1.6 ± 1.3 | 1.4 ± 1.0 | 1.5 ± 1.2 | 1.5 ± 1.2 |
| Milk, all types |  | 1.1 ± 1.0 | 0.8 ± 0.8 | 1.0 ± 0.9 | 1.2 ± 1.1 | 1.2 ± 1.1 |
| High fat milk, all types |  | 0.2 ± 0.5 | 0.1 ± 0.4 | 0.2 ± 0.4 | 0.2 ± 0.5 | 0.2 ± 0.5 |
| Low fat milk, all types |  | 0.9 ± 0.9 | 0.8 ± 0.8 | 0.9 ± 0.8 | 0.9 ± 1.1 | 1.0 ± 1.0 |
| Milk, regular |  | 0.9 ± 1.0 | 0.7 ± 0.8 | 0.9 ± 0.9 | 1.0 ± 1.1 | 1.1 ± 1.1 |
| High fat milk, regular |  | 0.1 ± 0.4 | 0.1 ± 0.3 | 0.1 ± 0.4 | 0.2 ± 0.5 | 0.1 ± 0.5 |
| Low fat milk, regular |  | 0.8 ± 0.9 | 0.6 ± 0.8 | 0.8 ± 0.8 | 0.9 ± 1.1 | 1.0 ± 1.0 |
| Fermented dairy |  | 2.2 ± 1.4 | 1.2 ± 1.1 | 1.5 ± 0.8 | 2.3 ± 0.9 | 3.7 ± 1.4 |
| High fat fermented dairy |  | 1.3 ± 1.2 | 0.1 ± 0.3 | 0.8 ± 0.4 | 1.4 ± 0.4 | 2.8 ± 1.1 |
| Low fat fermented dairy |  | 0.9 ± 0.9 | 1.1 ± 1.1 | 0.8 ± 0.8 | 0.9 ± 0.9 | 0.9 ± 0.9 |
| Yogurt |  | 0.5 ± 0.5 | 0.5 ± 0.5 | 0.5 ± 0.5 | 0.5 ± 0.5 | 0.6 ± 0.5 |
| High fat yogurt |  | 0.1 ± 0.3 | 0.1 ± 0.2 | 0.1 ± 0.3 | 0.2 ± 0.3 | 0.2 ± 0.4 |
| Low fat yogurt |  | 0.4 ± 0.5 | 0.4 ± 0.4 | 0.4 ± 0.5 | 0.4 ± 0.5 | 0.4 ± 0.5 |
| Cheese |  | 1.4 ± 1.1 | 0.6 ± 0.8 | 0.8 ± 0.4 | 1.4 ± 0.5 | 2.7 ± 1.1 |
| **High fat cheese** |  | 1.1 ± 1.1 | 0.1 ± 0.1 | 0.6 ± 0.2 | 1.2 ± 0.2 | 2.6 ± 1.1 |
| Low fat cheese |  | 0.2 ± 0.5 | 0.5 ± 0.8 | 0.2 ± 0.4 | 0.2 ± 0.4 | 0.1 ± 0.3 |
| Cream |  | 0.8 ± 2.5 | 0.6 ± 1.7 | 0.9 ± 2.4 | 1.0 ± 3.5 | 0.8 ± 2.1 |
| Ice cream |  | 0.06 ± 0.09 | 0.06 ± 0.10 | 0.06 ± 0.11 | 0.06 ± 0.09 | 0.06 ± 0.08 |
| **Dietary intake** |  |  |  |  |  |  |
| Energy intake (kcal) |  | 2100 ± 600 | 1900 ± 560 | 2100 ± 510 | 2200 ± 580 | 2400 ± 620 |
| DHD15-index score |  | 70 ± 14 | 71 ± 15 | 70 ± 14 | 70 ± 13 | 70 ± 13 |
| Fruit (g/day) |  | 200 ± 140 | 190 ± 140 | 190 ± 140 | 200 ± 150 | 200 ± 140 |
| Vegetables (g/day) |  | 150 ± 85 | 170 ± 89 | 150 ± 86 | 140 ± 78 | 140 ± 82 |
| Grain (g/day) | 5 | 200 ± 95 | 200 ± 94 | 190 ± 90 | 190 ± 93 | 210 ± 99 |
| Red meat (g/day) | 1 | 34 ± 23 | 36 ± 23 | 36 ± 23 | 32 ± 22 | 33 ± 23 |
| Processed meat (g/day) |  | 46 ± 33 | 38 ± 32 | 43 ± 30 | 50 ± 33 | 52 ± 35 |
| Lean fish (g/day) |  | 11 ± 13 | 11 ± 13 | 11 ± 12 | 11 ± 13 | 11 ± 14 |
| Fatty fish (g/day) |  | 5.0 ± 8.4 | 5.1 ± 7.7 | 5.2 ± 9.1 | 5.0 ± 8.8 | 4.6 ± 8.1 |
| Coffee (g/day) |  | 500 ± 270 | 460 ± 290 | 470 ± 270 | 530 ± 270 | 530 ± 250 |
| Tea (g/day) | 3 | 280 ± 260 | 280 ± 290 | 280 ± 250 | 270 ± 250 | 300 ± 270 |
| Fruit juice (g/day) | 3 | 60 ± 95 | 71 ± 110 | 59 ± 89 | 55 ± 94 | 54 ± 89 |
| Sugar-sweetened beverages (g/day) |  | 110 ± 140 | 120 ± 150 | 110 ± 140 | 110 ± 150 | 110 ± 140 |
| Saturated fat (en%) |  | 15 ± 3.7 | 12 ± 3.2 | 14 ± 3.2 | 16 ± 3.2 | 17 ± 3.4 |
| Protein (en%) |  | 15 ± 2.4 | 15 ± 2.8 | 14 ± 2.2 | 14 ± 2.3 | 15 ± 2.2 |
| Calcium (g) |  | 1000 ± 370 | 840 ± 320 | 910 ± 290 | 1000 ± 330 | 1300 ± 360 |
| Variables are displayed as means ± SD for normally distributed continuous variables, medians (IQR) for non-normally distributed continuous variables or % (n) for categorical variables.  BMI; Body Mass Index, DHD15-index; Dutch Healthy Diet 2015 index score [50], HS1; Hoorn Studies 1 (first enrolment wave), LDL; Low Density Lipoprotein. | | | | | | |

| **Supplemental Table 6.** *Sensitivity analyses for the association between dairy intake and incidence of prediabetes the Hoorn Studies.* | | | | | | |  |
| --- | --- | --- | --- | --- | --- | --- | --- |
|  | **Continuous^1^** | **Intake range categories** | | | |  | |
|  | RR (95%CI) |  | RR (95%CI) | RR (95%CI) | RR (95%CI) | P_trend_ | |
| **Total dairy** |  | **Q1** | **Q2** | **Q3** | **Q4** |  | |
| Main analysis model 3 | 0.98 (0.94-1.02) | 1 | 1.00 (0.86-1.16) | 0.87 (0.74-1.03) | 0.95 (0.79-1.13) | 0.38 | |
| 1. Adjustment other dairy | 0.97 (0.93-1.01) | 1 | 1.02 (0.88-1.18) | 0.87 (0.74-1.03) | 0.93 (0.78-1.11) | 0.26 | |
| 2. Excl. comorbidities | 0.98 (0.94-1.02) | 1 | 1.02 (0.86-1.19) | 0.89 (0.75-1.07) | 0.99 (0.82-1.20) | 0.76 | |
| 3. Exc. 'energy misreporters' | **0.95 (0.91-0.99)** | 1 | 1.09 (0.92-1.29) | 0.87 (0.71-1.05) | 0.93 (0.75-1.14) | 0.22 | |
| 4. Incl. baseline prediabetes | 0.98 (0.95-1.01) | 1 | 0.97 (0.85-1.10) | 0.91 (0.79-1.04) | 0.93 (0.80-1.08) | 0.30 | |
| **High fat dairy** |  | **Q1** | **Q2** | **Q3** | **Q4** |  | |
| Main analysis model 3 | 0.96 (0.91-1.01) | 1 | 1.01 (0.87-1.17) | 0.94 (0.79-1.11) | 0.85 (0.71-1.03) | 0.06 | |
| 1. Adjustment other dairy | 0.96 (0.91-1.01) | 1 | 1.01 (0.87-1.17) | 0.94 (0.79-1.11) | 0.85 (0.70-1.03) | 0.06 | |
| 2. Excl. comorbidities | 0.97 (0.92-1.02) | 1 | 1.00 (0.85-1.17) | 0.98 (0.82-1.17) | 0.90 (0.73-1.09) | 0.23 | |
| 3. Excl. 'energy misreporters' | 0.96 (0.90-1.01) | 1 | 0.93 (0.78-1.10) | 0.85 (0.70-1.03) | 0.85 (0.69-1.04) | 0.17 | |
| 4. Incl. baseline prediabetes | 0.96 (0.92-1.01) | 1 | 1.00 (0.88-1.14) | 0.94 (0.82-1.08) | 0.88 (0.75-1.03) | 0.08 | |
| **Low fat dairy** |  | **Q1** | **Q2** | **Q3** | **Q4** |  | |
| Main analysis model 3 | 1.01 (0.96-1.06) | 1 | 1.06 (0.91-1.23) | 1.01 (0.87-1.18) | 0.96 (0.82-1.13) | 0.48 | |
| 1. Adjustment other dairy | 1.00 (0.95-1.05) | 1 | 1.04 (0.89-1.21) | 0.98 (0.84-1.15) | 0.92 (0.78-1.09) | 0.25 | |
| 2. Excl. comorbidities | 1.00 (0.95-1.05) | 1 | 1.04 (0.89-1.23) | 1.02 (0.86-1.20) | 0.97 (0.81-1.15) | 0.62 | |
| 3. Excl. 'energy misreporters' | 0.97 (0.91-1.02) | 1 | 1.01 (0.84-1.21) | 1.03 (0.86-1.23) | 0.85 (0.70-1.03) | 0.12 | |
| 4. Incl. baseline prediabetes | 1.01 (0.97-1.05) | 1 | 1.03 (0.90-1.17) | 1.01 (0.88-1.15) | 0.95 (0.83-1.09) | 0.39 | |
| **Total fermented dairy** |  | **Q1** | **Q2** | **Q3** | **Q4** |  | |
| Main analysis model 3 | 0.98 (0.94-1.03) | 1 | 0.88 (0.75-1.03) | 0.95 (0.81-1.11) | 0.95 (0.80-1.13) | 0.81 | |
| 1. Adjustment other dairy | 0.98 (0.94-1.02) | 1 | 0.88 (0.75-1.03) | 0.95 (0.81-1.11) | 0.94 (0.80-1.12) | 0.73 | |
| 2. Excl. comorbidities | 0.99 (0.94-1.03) | 1 | 0.87 (0.74-1.03) | 0.95 (0.80-1.12) | 0.96 (0.80-1.15) | 0.86 | |
| 3. Excl. 'energy misreporters' | **0.95 (0.90-1.00)** | 1 | 0.81 (0.68-0.97) | 0.89 (0.74-1.06) | 0.86 (0.71-1.04) | 0.19 | |
| 4. Incl. baseline prediabetes | 0.98 (0.95-1.02) | 1 | 0.92 (0.81-1.05) | 0.93 (0.82-1.07) | 0.95 (0.83-1.10) | 0.61 | |
| **High fat fermented dairy** |  | **Q1** | **Q2** | **Q3** | **Q4** |  | |
| Main analysis model 3 | 0.95 (0.90-1.01) | 1 | **0.94 (0.80-1.09)** | **0.93 (0.79-1.08)** | **0.83 (0.69-0.99)** | **0.04** | |
| 1. Adjustment other dairy | 0.95 (0.90-1.01) | 1 | **0.94 (0.80-1.09)** | **0.93 (0.79-1.09)** | **0.83 (0.69-0.99)** | **0.04** | |
| 2. Excl. comorbidities | 0.96 (0.91-1.02) | 1 | 0.96 (0.81-1.13) | 0.94 (0.79-1.12) | 0.89 (0.74-1.08) | 0.25 | |
| 3. Excl. 'energy misreporters' | 0.94 (0.89-1.01) | 1 | **0.89 (0.75-1.07)** | **0.93 (0.78-1.11)** | **0.79 (0.65-0.97)** | **0.04** | |
| 4. Incl. baseline prediabetes | 0.97 (0.92-1.01) | 1 | 0.97 (0.85-1.11) | 0.93 (0.81-1.07) | 0.90 (0.78-1.05) | 0.17 | |
| **Low fat fermented dairy** |  | **Q1** | **Q2** | **Q3** | **Q4** |  | |
| Main analysis model 3 | 1.03 (0.97-1.10) | 1 | 0.99 (0.85-1.16) | 0.98 (0.84-1.15) | 0.96 (0.82-1.13) | 0.64 | |
| 1. Adjustment other dairy | 1.02 (0.96-1.09) | 1 | 0.99 (0.84-1.15) | 0.97 (0.83-1.14) | 0.94 (0.80-1.11) | 0.44 | |
| 2. Excl. comorbidities | 1.02 (0.96-1.09) | 1 | 1.00 (0.85-1.18) | 1.00 (0.84-1.18) | 0.94 (0.79-1.12) | 0.45 | |
| 3. Excl. 'energy misreporters' | 0.97 (0.91-1.05) | 1 | 1.00 (0.83-1.20) | 0.99 (0.82-1.18) | 0.90 (0.74-1.09) | 0.23 | |
| 4. Incl. baseline prediabetes | 1.01 (0.96-1.06) | 1 | 1.00 (0.88-1.14) | 0.97 (0.85-1.10) | 0.94 (0.82-1.08) | 0.33 | |
| **Total milk, all types** |  | **Q1** | **Q2** | **Q3** | **Q4** |  | |
| Main analysis model 3 | 1.00 (0.94-1.06) | 1 | 1.02 (0.87-1.19) | 1.13 (0.97-1.31) | 0.96 (0.81-1.15) | 0.79 | |
| 1. Adjustment other dairy | 1.00 (0.94-1.06) | 1 | 1.01 (0.87-1.19) | 1.12 (0.97-1.31) | 0.96 (0.81-1.14) | 0.76 | |
| 2. Excl. comorbidities | 1.00 (0.93-1.07) | 1 | 0.99 (0.84-1.18) | 1.15 (0.98-1.35) | 0.95 (0.79-1.15) | 0.85 | |
| 3. Excl. 'energy misreporters' | 0.98 (0.91-1.05) | 1 | 1.08 (0.90-1.30) | 1.21 (1.02-1.45) | 1.00 (0.81-1.23) | 0.79 | |
| 4. Incl. baseline prediabetes | 1.00 (0.95-1.05) | 1 | 1.04 (0.91-1.19) | 1.14 (1.00-1.29) | 1.00 (0.86-1.16) | 0.96 | |
| **High fat milk, all types** |  | **Zero** | **T1** | **T2** | **T3** |  | |
| Main analysis model 3 | 1.01 (0.89-1.14) | 1 | 0.91 (0.76-1.09) | 1.01 (0.82-1.24) | 1.04 (0.87-1.24) | 0.55 | |
| 1. Adjustment other dairy | 1.00 (0.88-1.13) | 1 | 0.91 (0.76-1.09) | 1.00 (0.81-1.24) | 1.02 (0.86-1.22) | 0.69 | |
| 2. Excl. comorbidities | 1.04 (0.91-1.18) | 1 | 0.92 (0.76-1.11) | 0.98 (0.78-1.23) | 1.06 (0.88-1.28) | 0.44 | |
| 3. Excl. 'energy misreporters' | 1.04 (0.92-1.18) | 1 | 0.89 (0.72-1.09) | 1.04 (0.82-1.33) | 1.10 (0.90-1.34) | 0.26 | |
| 4. Incl. baseline prediabetes | 1.00 (0.90-1.11) | 1 | 0.95 (0.81-1.10) | 1.00 (0.84-1.19) | 1.00 (0.87-1.16) | 0.88 | |
| **Low fat milk, all types** |  | **Q1** | **Q2** | **Q3** | **Q4** |  | |
| Main analysis model 3 | 0.99 (0.93-1.06) | 1 | 1.01 (0.86-1.19) | 1.04 (0.90-1.21) | 0.98 (0.83-1.17) | 0.88 | |
| 1. Adjustment other dairy | 0.99 (0.93-1.06) | 1 | 1.01 (0.86-1.19) | 1.04 (0.90-1.20) | 0.98 (0.82-1.16) | 0.80 | |
| 2. Excl. comorbidities | 0.99 (0.92-1.06) | 1 | 1.04 (0.88-1.24) | 1.08 (0.92-1.28) | 0.98 (0.82-1.16) | 0.72 | |
| 3. Excl. 'energy misreporters' | 0.96 (0.89-1.04) | 1 | 1.04 (0.86-1.24) | 1.02 (0.85-1.22) | 0.98 (0.80-1.18) | 0.73 | |
| 4. Incl. baseline prediabetes | 1.00 (0.95-1.05) | 1 | 1.02 (0.89-1.16) | 1.10 (0.96-1.25) | 0.97 (0.85-1.12) | 0.71 | |
| **Total regular milk** |  | **Q1** | **Q2** | **Q3** | **Q4** |  | |
| Main analysis model 3 | 1.00 (0.94-1.07) | 1 | 1.07 (0.91-1.26) | 1.11 (0.95-1.28) | 1.02 (0.85-1.24) | 0.79 | |
| 1. Adjustment other dairy | 1.00 (0.94-1.07) | 1 | 1.07 (0.91-1.26) | 1.11 (0.95-1.28) | 1.02 (0.84-1.23) | 0.81 | |
| 2. Excl. comorbidities | 1.01 (0.94-1.08) | 1 | 1.04 (0.88-1.24) | 1.17 (0.99-1.38) | 1.04 (0.88-1.24) | 0.73 | |
| 3. Excl. 'energy misreporters' | 0.98 (0.92-1.06) | 1 | 1.16 (0.95-1.41) | 1.12 (0.94-1.33) | 1.04 (0.84-1.30) | 0.79 | |
| 4. Incl. baseline prediabetes | 1.00 (0.95-1.06) | 1 | 1.05 (0.91-1.20) | 1.12 (0.98-1.27) | 1.04 (0.90-1.20) | 0.62 | |
| **High fat regular milk** |  | **Zero** | **T1** | **T2** | **T3** |  | |
| Main analysis model 3 | 1.00 (0.88-1.13) | 1 | 0.94 (0.69-1.27) | 1.18 (0.90-1.54) | 0.99 (0.75-1.31) | 0.81 | |
| 1. Adjustment other dairy | 0.99 (0.86-1.12) | 1 | 0.93 (0.69-1.25) | 1.15 (0.88-1.51) | 0.97 (0.74-1.28) | 0.96 | |
| 2. Excl. comorbidities | 1.02 (0.89-1.18) | 1 | 0.94 (0.68-1.30) | 1.20 (0.91-1.59) | 1.00 (0.74-1.35) | 0.75 | |
| 3. Excl. 'energy misreporters' | 1.04 (0.91-1.18) | 1 | 1.08 (0.79-1.46) | 1.30 (0.99-1.72) | 0.97 (0.70-1.36) | 0.72 | |
| 4. Incl. baseline prediabetes | 0.99 (0.89-1.11) | 1 | 0.94 (0.73-1.21) | 1.05 (0.82-1.35) | 1.02 (0.81-1.27) | 0.82 | |
| **Low fat regular milk** |  | **Q1** | **Q2** | **Q3** | **Q4** |  | |
| Main analysis model 3 | 1.01 (0.94-1.07) | 1 | 0.93 (0.79-1.09) | 1.10 (0.95-1.28) | 0.97 (0.82-1.13) | 0.87 | |
| 1. Adjustment other dairy | 1.00 (0.94-1.07) | 1 | 0.93 (0.79-1.09) | 1.10 (0.95-1.28) | 0.96 (0.81-1.12) | 0.78 | |
| 2. Excl. comorbidities | 1.00 (0.94-1.08) | 1 | 0.96 (0.81-1.14) | 1.12 (0.95-1.31) | 0.97 (0.82-1.16) | 0.92 | |
| 3. Excl. 'energy misreporters' | 0.97 (0.90-1.05) | 1 | 0.99 (0.83-1.19) | 1.02 (0.86-1.21) | 0.93 (0.75-1.17) | 0.63 | |
| 4. Incl. baseline prediabetes | 1.01 (0.95-1.06) | 1 | 0.98 (0.86-1.12) | 1.08 (0.95-1.23) | 0.96 (0.83-1.10) | 0.60 | |
| **Total yogurt** |  | **Q1** | **Q2** | **Q3** | **Q4** |  | |
| Main analysis model 3 | 1.04 (0.93-1.17) | 1 | 1.05 (0.89-1.23) | 1.02 (0.87-1.20) | 1.04 (0.88-1.21) | 0.73 | |
| 1. Adjustment other dairy | 1.04 (0.92-1.16) | 1 | 1.05 (0.89-1.23) | 1.02 (0.87-1.20) | 1.03 (0.88-1.21) | 0.77 | |
| 2. Excl. comorbidities | 1.05 (0.93-1.19) | 1 | 1.08 (0.91-1.28) | 1.05 (0.88-1.24) | 1.06 (0.89-1.26) | 0.59 | |
| 3. Excl. 'energy misreporters' | 0.99 (0.87-1.14) | 1 | 1.00 (0.83-1.20) | 0.95 (0.78-1.14) | 0.97 (0.81-1.17) | 0.68 | |
| 4. Incl. baseline prediabetes | 1.01 (0.91-1.11) | 1 | 1.07 (0.94-1.23) | 0.97 (0.85-1.12) | 1.04 (0.91-1.19) | 0.93 | |
| **High fat yogurt** |  | **Zero** | **T1** | **T2** | **T3** |  | |
| Main analysis model 3 | 1.15 (0.96-1.39) | 1 | 1.11 (0.90-1.36) | 1.15 (0.94-1.42) | 1.16 (0.95-1.42) | 0.10 | |
| 1. Adjustment other dairy | 1.08 (0.93-1.25) | 1 | 1.09 (0.93-1.28) | 1.20 (1.02-1.40) | 1.07 (0.91-1.25) | 0.21 | |
| 2. Excl. comorbidities | 1.16 (0.96-1.40) | 1 | 1.11 (0.91-1.34) | 1.21 (1.00-1.46) | 1.12 (0.92-1.36) | 0.14 | |
| 3. Excl. 'energy misreporters' | 1.15 (0.96-1.39) | 1 | 1.11 (0.90-1.36) | 1.15 (0.94-1.42) | 1.16 (0.95-1.42) | 0.10 | |
| 4. Incl. baseline prediabetes | 1.08 (0.93-1.25) | 1 | 1.09 (0.93-1.28) | 1.20 (1.02-1.40) | 1.07 (0.91-1.25) | 0.21 | |
| **Low fat yogurt** |  | **Zero** | **T1** | **T2** | **T3** |  | |
| Main analysis model 3 | 1.01 (0.89-1.14) | 1 | 1.04 (0.90-1.21) | 1.03 (0.87-1.21) | 0.99 (0.85-1.16) | 0.82 | |
| 1. Adjustment other dairy | 1.00 (0.89-1.13) | 1 | 1.04 (0.90-1.21) | 1.03 (0.87-1.21) | 0.99 (0.84-1.15) | 0.78 | |
| 2. Excl. comorbidities | 0.99 (0.87-1.13) | 1 | 1.04 (0.88-1.22) | 1.04 (0.88-1.24) | 0.96 (0.81-1.13) | 0.65 | |
| 3. Excl. 'energy misreporters' | 0.92 (0.78-1.07) | 1 | 1.00 (0.84-1.19) | 0.98 (0.81-1.19) | 0.91 (0.76-1.10) | 0.31 | |
| 4. Incl. baseline prediabetes | 0.97 (0.88-1.08) | 1 | 1.02 (0.90-1.15) | 1.00 (0.87-1.15) | 0.95 (0.83-1.08) | 0.43 | |
| **Total cheese** |  | **Q1** | **Q2** | **Q3** | **Q4** |  | |
| Main analysis model 3 | 0.95 (0.90-1.01) | 1 | **1.02 (0.88-1.19)** | **0.90 (0.77-1.05)** | **0.86 (0.73-1.02)** | **0.04** | |
| 1. Adjustment other dairy | 0.95 (0.90-1.01) | 1 | **1.02 (0.88-1.19)** | **0.90 (0.77-1.05)** | **0.86 (0.73-1.03)** | **0.04** | |
| 2. Excl. comorbidities | 0.96 (0.91-1.01) | 1 | 0.97 (0.83-1.14) | 0.87 (0.74-1.03) | 0.87 (0.73-1.04) | 0.09 | |
| 3. Excl. 'energy misreporters' | **0.92 (0.87-0.99)** | 1 | **1.00 (0.84-1.19)** | **0.87 (0.73-1.04)** | **0.78 (0.64-0.96)** | **0.01** | |
| 4. Incl. baseline prediabetes | 0.97 (0.92-1.01) | 1 | 1.00 (0.88-1.13) | 0.87 (0.76-1.00) | 0.90 (0.78-1.04) | 0.07 | |
| **High fat cheese** |  | **Q1** | **Q2** | **Q3** | **Q4** |  | |
| Main analysis model 3 | **0.94 (0.88-1.00)** | 1 | **0.95 (0.82-1.10)** | **0.91 (0.77-1.07)** | **0.79 (0.66-0.94)** | **0.01** | |
| 1. Adjustment other dairy | **0.94 (0.88-1.00)** | 1 | **0.95 (0.82-1.10)** | **0.91 (0.77-1.07)** | **0.79 (0.66-0.94)** | **0.01** | |
| 2. Excl. comorbidities | 0.95 (0.89-1.01) | 1 | 0.96 (0.82-1.13) | 0.96 (0.81-1.14) | 0.84 (0.70-1.02) | 0.07 | |
| 3. Excl. 'energy misreporters' | **0.92 (0.86-0.99)** | 1 | **0.90 (0.76-1.07)** | **0.87 (0.73-1.04)** | **0.73 (0.59-0.90)** | **0.003** | |
| 4. Incl. baseline prediabetes | 0.96 (0.91-1.00) | 1 | **0.95 (0.83-1.08)** | **0.94 (0.82-1.08)** | **0.85 (0.74-0.98)** | **0.03** | |
| **Low fat cheese** |  | **Zero** | **T1** | **T2** | **T3** |  | |
| Main analysis model 3 | 1.04 (0.94-1.16) | 1 | 1.03 (0.85-1.25) | 0.99 (0.82-1.19) | 1.12 (0.93-1.34) | 0.30 | |
| 1. Adjustment other dairy | 1.04 (0.94-1.15) | 1 | 1.01 (0.83-1.23) | 0.98 (0.81-1.18) | 1.10 (0.92-1.32) | 0.37 | |
| 2. Excl. comorbidities | 1.02 (0.92-1.14) | 1 | 0.92 (0.75-1.15) | 0.92 (0.75-1.12) | 1.06 (0.87-1.29) | 0.66 | |
| 3. Excl. 'energy misreporters' | 0.97 (0.86-1.10) | 1 | 1.04 (0.83-1.29) | 0.91 (0.72-1.15) | 1.02 (0.82-1.27) | 0.95 | |
| 4. Incl. baseline prediabetes | 1.03 (0.95-1.12) | 1 | 1.00 (0.84-1.19) | 1.00 (0.85-1.17) | 1.08 (0.92-1.25) | 0.39 | |
| **Cream** |  | **Zero** | **T1** | **T2** | **T3** |  | |
| Main analysis model 3 | 0.98 (0.96-1.01) | 1 | 1.02 (0.86-1.21) | 0.89 (0.76-1.05) | 0.87 (0.74-1.03) | 0.08 | |
| 1. Adjustment other dairy | 0.96 (0.92-1.01) | 1 | 1.02 (0.86-1.21) | 0.89 (0.76-1.05) | 0.87 (0.72-1.05) | 0.11 | |
| 2. Excl. comorbidities | 0.98 (0.95-1.01) | 1 | 1.03 (0.86-1.23) | 0.89 (0.75-1.05) | 0.85 (0.71-1.02) | 0.05 | |
| 3. Excl. 'energy misreporters' | 0.98 (0.95-1.01) | 1 | 1.01 (0.83-1.24) | 0.89 (0.74-1.06) | 0.85 (0.70-1.03) | 0.07 | |
| 4. Incl. baseline prediabetes | 0.99 (0.97-1.01) | 1 | 0.96 (0.83-1.12) | 0.89 (0.78-1.02) | 0.91 (0.79-1.05) | 0.21 | |
| **Ice cream** |  | **Zero** | **T1** | **T2** | **T3** |  | |
| Main analysis model 3 | 0.79 (0.42-1.48) | 1 | 1.06 (0.87-1.28) | 0.99 (0.86-1.13) | 0.95 (0.81-1.12) | 0.44 | |
| 1. Adjustment other dairy | 0.76 (0.40-1.43) | 1 | 1.05 (0.87-1.28) | 0.98 (0.86-1.12) | 0.94 (0.80-1.11) | 0.39 | |
| 2. Excl. comorbidities | 0.78 (0.39-1.56) | 1 | 1.08 (0.88-1.33) | 1.01 (0.88-1.17) | 0.99 (0.83-1.18) | 0.76 | |
| 3. Excl. 'energy misreporters' | 0.74 (0.36-1.55) | 1 | 1.10 (0.87-1.38) | 1.00 (0.86-1.18) | 0.99 (0.82-1.20) | 0.78 | |
| 4. Incl. baseline prediabetes | 0.73 (0.42-1.29) | 1 | 0.96 (0.82-1.14) | 0.94 (0.84-1.05) | 0.93 (0.82-1.07) | 0.31 | |
| ^1^ Continuous analyses in servings/day: milk, 200 ml; yogurt, 150 ml; cheese, 20 g; cream 3 g; ice cream, 100 g. Combined total dairy category: liquid dairy products, 200 ml; cheese, 20g.  Model 3 included age (continuous), sex, follow-up duration, cohort, energy intake (continuous), education (3 categories), smoking (3 categories), physical activity (continuous), alcohol consumption (4 categories), family history of diabetes (yes/no), intakes of fruit, vegetables, tea, coffee, grains (whole and refined), meat (processed and red) and sugar-sweetened beverages (continuous). HS, Hoorn Study; Q, quartile; RR, Risk Ratio; T, Tertile. Significant associations are indicated in **Bold.** | | | | | | |  |

| **Supplemental table 7.** *Sensitivity analyses for the substitution of high fat cheese with alternative dairy products and incidence of prediabetes the Hoorn Studies^1^* | | | |
| --- | --- | --- | --- |
|  | Exclusion comorbidities  *n*=*2,201* | Energy normal reported only  *n*=1,716 | Inclusion prediabetes at baseline *n*=2,661 |
|  | RR (95%CI) | RR (95%CI) | RR (95%CI) |
| **High fat cheese** |  |  |  |
| High fat milk, all types | 1.04 (0.90-1.21) | 1.09 (0.94-1.26) | 1.06 (0.93-1.21) |
| Low fat milk, all types | 1.04 (0.94-1.14) | 1.05 (0.94-1.17) | 1.06 (0.96-1.16) |
| High fat yogurt | 1.22 (0.99-1.49) | 1.23 (1.00-1.52) | 1.20 (0.99-1.46) |
| Low fat yogurt | 1.05 (0.90-1.23) | 1.02 (0.85-1.23) | 1.09 (0.95-1.26) |
| Low fat cheese | 1.06 (0.94-1.20) | 1.06 (0.93-1.21) | 1.09 (0.98-1.22) |
| Cream | 1.02 (0.95-1.09) | 1.06 (0.98-1.15) | 1.05 (0.98-1.12) |
| Ice cream | 0.84 (0.43-1.62) | 0.85 (0.42-1.73) | 0.82 (0.44-1.54) |
| ^1^ Continuous analyses in servings/day: milk, 200 ml; yogurt, 150 ml; cheese, 20 g; cream 3 g; ice cream, 100 g. Substitution models included total servings/day of dairy intake, energy intake (kcal).  Models were adjusted for age (continuous), sex, follow-up duration, cohort, education (3 categories), smoking (3 categories), physical activity (continuous), alcohol consumption (4 categories), family history of diabetes (yes/no), intakes of fruit, vegetables, tea, coffee, grains (whole and refined), meat (processed and red) and sugar-sweetened beverages (continuous). HS, Hoorn Study; Q, quartile; RR, Risk Ratio. Significant associations are indicated in **Bold.** | | | |
